# Identification and therapeutic modulation of a pro-inflammatory subset of disease-associated-microglia in Alzheimer’s disease

**DOI:** 10.1186/s13024-018-0254-8

**Published:** 2018-05-21

**Authors:** Srikant Rangaraju, Eric B. Dammer, Syed Ali Raza, Priyadharshini Rathakrishnan, Hailian Xiao, Tianwen Gao, Duc M. Duong, Michael W. Pennington, James J. Lah, Nicholas T. Seyfried, Allan I. Levey

**Affiliations:** 1 Department of Neurology, Emory University, Atlanta, GA 30322 USA; 2 Department of Biochemistry, Emory University, Atlanta, GA 30322 USA; 3 Emory University, Atlanta, GA 30322 USA; 4 Peptides International, Louisville, KY 40269 USA

**Keywords:** Microglia, Macrophage, Alzheimer’s disease, Network analysis, Kv1.3, Potassium channel, Amyloid, Neuroinflammation

## Abstract

**Background:**

Disease-associated-microglia (DAM) represent transcriptionally-distinct and neurodegeneration-specific microglial profiles with unclear significance in Alzheimer’s disease (AD). An understanding of heterogeneity within DAM and their key regulators may guide pre-clinical experimentation and drug discovery.

**Methods:**

Weighted co-expression network analysis (WGCNA) was applied to existing microglial transcriptomic datasets from neuroinflammatory and neurodegenerative disease mouse models to identify modules of highly co-expressed genes. These modules were contrasted with known signatures of homeostatic microglia and DAM to reveal novel molecular heterogeneity within DAM. Flow cytometric validation studies were performed to confirm existence of distinct DAM sub-populations in AD mouse models predicted by WGCNA. Gene ontology analyses coupled with bioinformatics approaches revealed drug targets and transcriptional regulators of microglial modules predicted to favorably modulate neuroinflammation in AD. These guided in-vivo and in-vitro studies in mouse models of neuroinflammation and neurodegeneration (5xFAD) to determine whether inhibition of pro-inflammatory gene expression and promotion of amyloid clearance was feasible. We determined the human relevance of these findings by integrating our results with AD genome-wide association studies and human AD and non-disease post-mortem brain proteomes.

**Results:**

WGCNA applied to microglial gene expression data revealed a transcriptomic framework of microglial activation that predicted distinct pro-inflammatory and anti-inflammatory phenotypes within DAM, which we confirmed in AD and aging models by flow cytometry. Pro-inflammatory DAM emerged earlier in mouse models of AD and were characterized by pro-inflammatory genes (Tlr2, Ptgs2, Il12b, Il1b), surface marker CD44, potassium channel Kv1.3 and regulators (NFkb, Stat1, RelA) while anti-inflammatory DAM expressed phagocytic genes (Igf1, Apoe, Myo1e), surface marker CXCR4 with distinct regulators (LXRα/β, Atf1). As neuro-immunomodulatory strategies, we validated LXRα/β agonism and Kv1.3 blockade by ShK-223 peptide that promoted anti-inflammatory DAM, inhibited pro-inflammatory DAM and augmented Aβ clearance in AD models. Human AD-risk genes were highly represented within homeostatic microglia suggesting causal roles for early microglial dysregulation in AD. Pro-inflammatory DAM proteins were positively associated with neuropathology and preceded cognitive decline confirming the therapeutic relevance of inhibiting pro-inflammatory DAM in AD.

**Conclusions:**

We provide a predictive transcriptomic framework of microglial activation in neurodegeneration that can guide pre-clinical studies to characterize and therapeutically modulate neuroinflammation in AD.

**Electronic supplementary material:**

The online version of this article (10.1186/s13024-018-0254-8) contains supplementary material, which is available to authorized users.

## Background

Microglia represent innate immune cells of the CNS that play important disease-modifying roles in neurodegeneration including Alzheimer’s disease (AD) [1–4]. The importance of microglia-mediated neuroinflammation in neurodegeneration has been confirmed by genetic studies that identified several immune gene polymorphisms as risk factors for AD [5–8]. Data from mouse models have suggested dual roles for microglia in AD: pro-inflammatory functions that promote neurotoxicity and amyloid β (Aβ) accumulation, opposed by amyloid-clearing and neuroprotective functions [3, 4]. Recent transcriptomics studies have shown that homeostatic microglia gradually adopt a unique phagocytic disease-associated microglia (DAM) phenotype in neurodegenerative disease, chronic neuroinflammatory states as well as advanced aging [9–12]. While single-cell transcriptomic studies have not clarified molecular or functional heterogeneity within DAM [9], meta-analyses and network-based approaches applied to deeper bulk microglial transcriptomes have indicated greater diversity within microglia in neurodegeneration [10, 13]. Based on analysis of microglial transcriptomic data, the transcriptional signature of “primed” microglia from neurodegenerative disease brains is suggestive of increased phago-lysosome, oxidative phosphorylation and antigen-presentation functions [12, 13]. Trem2, a myeloid protein involved in microglial survival and proliferation, regulates a checkpoint necessary for DAM and deletion of Trem2 prevents microglial accumulation around Aβ plaques and leads to additional neuritic damage [6, 14–17]. These findings suggest that DAM may be protectively geared towards more effective phagocytosis and clearance of pathological protein aggregates in neurodegenerative disorders. However, global microglial depletion in AD mouse models resulted in a protective effect on synaptic health, independent of amyloid β [18, 19]. These emphasize the immense complexity of microglial functional roles in neurodegeneration [20], and support the existence of distinct pro-inflammatory functional states within DAM. A recent network analysis of bulk transcriptomes identified distinct microglial co-expression networks (or modules) which when applied to re-analyze microglial single-cell RNAseq data [9, 10] identified novel interferon-related and lipopolysaccharide (LPS)-related co-expression modules and previously unappreciated microglial sub-populations, in addition to DAM in neurodegeneration models [10]. This highlights the value of integrating deeper bulk transcriptomic findings with single cell data to maximize the chances of identifying cellular heterogeneity within a cell population.

A comprehensive understanding of key regulators, markers and drug targets of homeostatic, pro-inflammatory and anti-inflammatory microglial subtypes could provide novel biological insights and facilitate target nomination and prioritization of immunomodulatory therapeutic approaches in AD [21, 22]. In this study, we applied weighted correlation network analysis (WGCNA) to existing transcriptomic microarray datasets obtained from purified CD11b^+^ CNS immune cells spanning neuroinflammatory and neurodegenerative disease states [15, 22, 23] to first identify distinct modules of co-expressed microglial genes that are associated with AD pathology. We then mapped these AD-specific gene modules to existing microglial single-cell RNAseq data to determine whether WGCNA modules represented distinct microglial populations and to further identify molecular heterogeneity within DAM [9]. Gene member and ontological analyses of modules coupled with bioinformatics approaches (connectivity map analysis) revealed specific drug targets and transcriptional regulators of AD-specific microglial modules (LXRα/β for anti-inflammatory DAM and Kv1.3 for pro-inflammatory DAM) as well as flow cytometric markers for each DAM subtype. Flow cytometric studies informed by WGCNA results confirmed distinct pro-inflammatory and anti-inflammatory subpopulations and suggested additional heterogeneity within DAM in aging and the 5xFAD model of AD pathology [24]. In-vivo pharmacologic studies using LXRα/β agonists and Kv1.3 channel inhibitors were then performed to determine whether modulation of pro- and anti-inflammatory DAM gene expression, proportions of DAM subsets and Aβ plaque pathology in 5xFAD mice could be achieved. Finally, we determined the relevance of homeostatic and DAM modules in human AD by integrating our findings with existing AD genome-wide associated studies (GWAS) as well as a human post-mortem brain proteomes from AD and non-disease controls.

## Methods

### Reagents

ShK-223 peptide was synthesized and folded as previously described [25]. The IC_50_ of ShK-223 for Kv1.3 channels is 25 ± 14 pM, > 10,000 times more selective for Kv1.3 channels as compared to neuronal Kv1.1 and Kv1.2 channels [25]. ShK-223 used for in-vitro at 100 nM concentration and 100 μg/kg for in-vivo (IP) injections in mice [26]. A validated fluorescein-conjugated ShK analog (ShK-F6CA) was used to detect functional cell surface Kv1.3 channels in microglia (Peptides International (Louisville, Kentucky) [26, 27]. LPS was obtained from Sigma Aldrich (Cat. #L4391, *Escherichia coli* 0111:B4). LXRα/β agonist T0901317 was obtained from Cayman Chemicals (Cat# 71810) and dissolved in DMSO and was diluted in saline prior to administration.

### Flow cytometric and immunohistochemistry antibodies

Monoclonal fluorophore-conjugated antibodies used for flow cytometry were obtained from BD Biosciences (CD11b-APC-Cy7, CD45-PeCy7, CD11c-BV421, CD274-APC, CD69-APC, CD44-PeCy7 or FITC, CD184-PE, CD3e-FITC, Ly-6c-PE, Ly-6G-APC) and BioLegend (CD8a-BV785, CD45-PerCP, and ENPP1-BV421). For immunohistochemistry, rabbit anti-Iba1 monoclonal antibody (Abcam # ab178846) was used at 1: 500 dilution. Rat anti-mouse LAMP1 monoclonal Ab was obtained from the Hybridoma Bank (Cat # 1D4B) and used at 1: 250 dilution. Mouse anti-CD68 monoclonal Ab (Abcam # ab955) was used at 1: 200 dilution. To identify amyloid plaques, anti-Aβ monoclonal 4G8 (Aβ 17–42) antibody (Signet Cat # 9220–02) was used at 1: 1000 dilution. Secondary anti-mouse, anti-goat and anti-rabbit antibodies (Jackson labs and Sigma-Aldrich) were used for immunohistochemistry. In immunofluorescence studies, Rhodamine-conjugated donkey anti-rat IgG (1: 500), Alexa-488-conjugated goat anti-rabbit (1: 500), and DyLight 405-conjugated donkey anti-mouse (1: 500) were used as secondary antibodies. Fluorescent-labeled Aβ_42_ fibrils for immunofluorescence studies were prepared as described below and FITC filter was used to observe Aβ fluorescence.

### Primary microglial isolation

Adult C57BL6 mice, age-matched female 5xFAD mice were euthanized and brains were isolated following rapid cold saline cardiac perfusion and CNS immune cells were isolated as previously described [26, 28]. Briefly, brains were minced over a 40 μm cell strainer and single cell suspensions were washed in PBS in a centrifuge for 5 min at 800×g at room temperature. Supernatants were discarded and cell pellets re-suspended in 6 mL of 37% stock isotonic Percoll (SIP) solution (90% Percoll + 10% 10X HBSS) per brain. The cell suspension was transferred into 15 mL conical tubes and 2 mL of 70% SIP slowly under laid. Then on top of the 37% layer, 2 mL of 30% SIP was slowly layered. The established gradient was then centrifuged for 25 min at 800×g with zero deceleration at 20 °C. Floating myelin in the top layer was then removed and a Pasteur pipette used to carefully collect 3 mL from the 70–37% interphase without disturbing the 70% layer. Cells were then washed × 3 in 10 mL cold PBS and the pellets comprising of CNS immune cells were then re-suspended in 100 μL of the appropriate buffer.

### Microglial Aβ42 phagocytosis assay

The ability of acutely isolated CNS mononuclear cells from wild type, LPS-treated and age-matched 5xFAD mice to phagocytose fluorescent-labeled Aβ fibrils ex-vivo was assessed by a flow cytometric assay. Fluorescent-labeled Aβ42 fibrils were prepared by co-incubating unlabeled and Hilyte488-labeled Aβ42 required unlabeled Aβ42 (Anaspec Cat # AS-60479-01) at a 1:4 ratio. Purified Aβ42 was first linearized in 10% *w*/*v* NH_4_OH and re-lyophilized to reduce any preexisting aggregates. 0.5 mg of unlabeled lyophilized Aβ42 was then reconstituted to 150 μM by adding 1 mM NaOH, bath sonicated for 10 min followed by addition of 10X buffer (100 mM sodium phosphate, pH 7.1) to bring the final pH to 7.4. 410.64 μL of that aliquot was added to 0.1 mg of HiLyte-488 Aβ42 to achieve a total of 200 μM Aβ42 solution. The sample was incubated in dark at room temperature for 5–7 days before being transferred to 4 °C. Phagocytosis of fluorescent-labeled Aβ42 fibrils (fAβ42-HiLyte488) was performed by incubating acutely isolated CNS immune cells (pretreated ex-vivo with ShK-223 at 100 nM or vehicle) with the reagent at 2.5 μM for 30 min at 37 °C in a 5% CO_2_ humidified incubator. The cells were then washed with cold PBS and labeled with fluorophore-conjugated CD11b (CD11b-APC-Cy7, BD Biosciences Cat # 557657) and CD45 (CD45-PerCP, BioLegend Cat # 103130) for 30 min at 4 °C. This was followed by washing with cold flow cytometry buffer prior to flow cytometry [29, 30]. Compensation experiments were performed prior to performing the phagocytosis assay using compensation beads. Before sample data acquisition, unstained sample was run (without fAβ42-HiLyte488) and then positive control was run at low speed to allow for voltage level adjustment considering the high intensity of Aβ42 fluorescence in Alexa-488 channel. For phagocytosis assay using flow cytometry, CNS immune cells were first gated for live cells using FSC/SSC gating followed by gating for singlets using FSC-A/FSC-H. Following selection of singlets, CD11b and CD45 gating was used to identify CD11b^+^, CD11b^+^CD45^low^ and CD11b^+^CD45^high^ populations after which phagocytosis was assessed in each group using the second peak of Aβ fluorescence as recently described [31]. We have found that Cytochalasin D inhibits the second peak of fluorescence in this assay, suggesting that the first peak represent non-specific binding of Aβ42 to cells while the second peak represents true uptake in an actin-dependent manner [31].

### Animals

Female C57BL/6 J and female 5xFAD mice used for the studies were housed in the Department of Animal Resources at Emory University under standard conditions with no special food/water accommodations. Institutional Animal Care and Use Committee approval was obtained prior to in-vivo work and all work was performed in strict accordance with the Guide for the Care and Use of Laboratory Animals of the National Institutes of Health. Adult mice were given intraperitoneal LPS injections (10 μg/dose × 4 daily doses) to induce acute neuroinflammation [26, 32]. If ≥25% of weight loss was observed, animals were euthanized. In some experiments, 5xFAD mice received i.p. doses of T0901317 (30 mg/kg) twice a week for 2 weeks.

### GEO datasets, microarray normalization, and batch correction

Microarray transcriptomic datasets were obtained from the genomics data repository Gene Expression Omnibus (GEO). Microarray transcriptome datasets of FACS-purified microglia from 8.5 mo old WT (*n* = 5), TREM2 −/− (*n* = 4), 5xFAD (n = 5) and TREM2 ^−/−^/5xFAD (*n* = 6) mice were obtained from GEO (GSE65067). GEO dataset GSE49329 provided microarray data from murine microglia treated in-vitro with PBS (control), LPS or IL4 (*n* = 9 arrays). These two disparate Affymetrix platform datasets were RMA normalized using the affy package RMA function, combined, and corrected for batchwise artefacts using WT and PBS-treated microglia as comparable control features and other samples as experimental features across the 2 batches specified as such to the ComBat algorithm loaded from the R SVA package. Unique batch-corrected array features representing gene-level expression were then selected using a filter to retain features with maximum variance across the 29 samples using the WGCNA collapseRows function. Notably, the array annotations for mogene 1.0 database package were downloaded as of September 2015, and this package version is provided to make results, in particular the intersection of common genes across the arrays, reproducible. Existing RNAseq microglial transcriptomes were not included due to lower depth of coverage and inability to sufficiently overcome batch effects when combining microarray and RNAseq transcriptomes.

Agilent microarray data from microglia isolated from WT and APP/PS1 mice (*n* = 7 each) were obtained as a subset of the samples in GEO dataset GSE74615. Agilent green (Cy3) and red (Cy5) channels were background corrected according to the normexp method in limma::backgroundCorrect() function, [33] then quantile-normalized. Genes expressed on average at greater than 400% of the 95th percentile intensity of negative control probes were considered as expressed. Log_2_(Cy5/Cy3) signals were calculated and a histogram calculation of shift from 0 was used to center the Gaussian of intensities across the 2 channels. Then WGCNA::collapseRows was applied as above, and astrocyte-specific measurements (*n* = 8/22) were removed. 16,721 gene symbols shared between the expressed genes measured on Affymetrix and Agilent platforms were found and the corrected, normalized, log2-transformed expression values for each platform were combined into a single matrix and subjected to a second pass of SVA::comBat(). The batch-corrected, adjusted datasets were then used for WGCNA. R code is provided online, via synapse.org (https://www.synapse.org/#!Synapse:syn10934660).

In a separate replication analysis, transcript count data from 64 purified microglial medium-throughput sequencing (Nanostring) studies (GEO dataset GSE101689) were obtained from supplemental data [16] which were log2-transformed (0 counts set to 0.5) and batch-corrected using ComBat and assumption of common samples across batches represented by WT or PBS/sham treated mice, considering EAE, LPS, APP/PS1 and SOD datasets as separate batches. Three hundred and sixty-nine genes that were identified across all datasets without any missing data were used for WGCNA as described below. Effectiveness of batch correction was confirmed by comparing the consistency of geometric mean of batch-corrected counts for the 6 pre-determined housekeeping genes in different Nanostring nCounter assays.

### WGCNA

The R package WGCNA was used to construct a co-expression network using normalized and batch-corrected data of the 43 microarray-measured samples just described using a previously published approach [34]. A threshold power of 10 was chosen since it was the smallest threshold that resulted in a scale-free R^2^ fit of 0.8. The network was created using WGCNA::blockwiseModules() function, in a single block (maxBlockSize > 16,721). Briefly, this function calculated topologic overlap (TO) with bicor correlation function, then genes were hierarchically clustered using 1-TO (dissTOM) as the distance measure. Initial module assignments were determined by using dynamic tree-cutting, using default parameters except deepSplit = 2, mergeCutHeight =0.20, minModulesize = 40, pamStage = TRUE, and pamRespectsDendro = TRUE), but genes were allowed to be reassigned to modules with better correlation if *p* < 0.05 for those correlations. The network type was signed, so that anticorrelated genes were not assigned to the same module. Resulting 19 modules or groups of co-expressed genes ranging in size from 2165 genes (turquoise) to 75 genes (lightyellow) were used to calculate the eigengenes (MEs; or the 1st principal component of the module). MEs were correlated with different biological traits including AD (model), LPS-treated and IL4-treated. R code is provided online via synapse.org (https://www.synapse.org/#!Synapse:syn10934660).

In the validation WGCNA, scale free topology was achieved with beta (power) set at 11.5, and other blockwiseModules() function parameters were deepSplit = 4, minModulesize = 3, mergeCutHeight = 0.12, pamStage = TRUE, pamRespectsDentro = TRUE, reassignThresh = 0.05. Over-representation analysis (ORA) was performed with in an house custom script testing for hypergeometric overlap of gene symbol membership in modules across the derivation and nanostring (validation) networks using the fisher.test() function, with one-tailed sensitivity for overrepresentation of module membership, i.e. using alternative = “greater” parameter.

### viSNE plots

WGCNA network gene-level k_ME_ values (gene expression correlation to the module eigengene for the module to which each gene belonged) were used to cull the measured gene list to genes with k_ME_ > 0.65 and membership in any of 8 modules of particular interest. Then, normalized, corrected array log2 expression data was row-normalized to set the sample average (row mean) for each gene to zero. Rtsne R package Rtsne Barnes-Hut-Stochastic Neighbor Embedding (SNE) function was then applied to the culled 43-dimension data matrix of genes, reducing it to a 2-dimensional projection for each gene [35] vi-SNE output points representing genes were then colored by the WGCNA module membership of each gene. In another version of the projection, membership of the DAM-microglial genes and Homeostatic microglial genes were also mapped onto the identical vi-SNE projection points. DAM and Homeostatic genes were defined based on published single cell RNAseq data. DAM-specific genes were defined as ≥4-fold higher expression in DAM cells while Homeostatic genes were defined as ≥4-fold higher expression in Homeostatic microglia as compared to DAM cells [9]. R code is provided online via synapse.org (https://www.synapse.org/#!Synapse:syn10934660).

### MAGMA GWAS target module over-representation analysis

MAGMA for AD IGAP GWAS targets (*N* = 50,000 patients) and subsequent over-representation analysis for module membership of GWAS target-genes using statmod function permutation was performed as previously published [34]. MAGMA script output for gene-wise *p*-values for AD and other conditions for which GWAS SNPs have been reported selected from the UCSC Genome Browser GWAS track are provided with the R code online at synapse.org (https://www.synapse.org/#!Synapse:syn10934660).

### In-vitro assays of fAβ42 degradation and reactive oxygen species production by microglia

Bv2 microglia were maintained in Dulbecco’s modified Eagle Medium (with 10% FBS). For in-vitro experiments, Bv2 cells were grown on glass cover slips, loaded with fluorescent Aβ42 fibrils and cultured up to 72 h. At different time points 0.5, 6, 16, 24, 48 and 72 h, the BV2 microglia were fixed for 15 min, washed gently with PBS, permeabilized and mounted in Vectashield hard set mounting medium containing DAPI followed by image acquisition. In separate experiments, Bv2 microglia were first loaded with fluorescent Aβ42 for 1 h, washed × 3, and incubated with 100 nM of ShK-223 and then maintained in culture for 0.5, 6, 16, 24, 48 and 72 h. At each time point, cells were fixed, washed, permeabilized with 1X Permeabilization buffer (eBioscience Cat # 00–8333-56) and then washed with PBS. This was followed by incubation with blocking buffer (10% normal horse serum in PBS) for 30 min and incubation with rat anti-mouse LAMP1 monoclonal antibody and then the appropriate fluorophore-conjugated secondary antibody. This was then followed by image acquisition for co-localization analyses. Co-localization analysis was performed using the co-localization threshold plugin in ImageJ (Fiji image analysis software Version 1.51n). Green and Red channel images at each time point were converted to gray-scale in FIJI, and co-localization threshold plugin was applied to the region of interest that was limited to one cell at a time.

### Immunofluorescence microscopy

Double antigen immunofluorescence for Aβ and Iba1 in slides from ShK-223 and PBS treated mice involved antigen retrieval with 45% formic acid for 5 min followed by heating in microwave for 10 min in 10 mM citric acid (pH 6.0) solution. This was followed by similar blocking steps as outlined above and then overnight incubation in anti-Aβ (4G8) and anti-Iba1 primary antibodies. DyLight 405-conjugated donkey anti-mouse IgG (1: 500) and Alexa-Fluor488-conjugated goat anti-rabbit IgG (1: 500) were used as secondary antibodies. Single antibody controls for each channel were used to optimize settings. Fluorescent images were obtained with a fluorescence microscope (Microscope: Olympus BX51 and Camera: Olympus DP70) using FITC, PE and DAPI filters and the images were processed for further analyses. Immunofluorescence microscopy was also performed for assessment of LAMP-1 and fAβ42 co-localization in mature phago-lysosomes in BV2 microglia. LAMP1 was labeled with rat anti-mouse LAMP1 monoclonal primary antibody followed by anti-rat Rhodamine-conjugated IgG (1: 500). The Bv2 cells on coverslips from each time point after being immunostained were mounted in Vectashield hard set mounting medium containing DAPI and at least 10 images per condition, each in triplicate (acquired at 40× magnification), were collected.

### Flow cytometric studies

Freshly isolated CNS immune cells were labeled for CD11b, CD45, CD11c, CD44, CD184 (CXCR4), CD69 and CD274 using well characterized fluorophore-conjugated monoclonal antibodies. Kv1.3 channels expression on cell surface was assessed using fluorescein-conjugated ShK analog as stated above. Compensation experiments were run using compensation beads prior to sample data acquisition using previously published protocols [26]. FSC/SSC gating was used to establish live cell population followed by FSC-A/FSC-H gating to gate for single cells. CD11b and CD45 were then used to identify and gate for CD11b^+^CD45^low^ and CD11b^+^CD45^high^ populations with the criterion for level of CD45^high^ set on the basis of CD45 expression in CD11b-CD3 + CD45^high^ lymphocyte population. CD11b^+^CD45^low^ and CD11b^+^CD45^high^ gating strategy was also employed for the ex-vivo phagocytosis assay. In separate experiments, CNS immune cells and splenocytes were also labeled for Ly6c and Ly6G using fluorophore-conjugated antibodies.

### Immunohistochemistry

For all these studies, three sagittal brain sections (one from each treatment group and from WT mice) from equivalent regions were placed on each slide to control for any heterogeneity in staining. Paraffin embedded sections of brains obtained from mice from the ShK-223 treatment trial were de-paraffinized in Histoclear and rehydrated, incubated in 90% formic acid for 10 min, washed in buffer, blocked with 3% hydrogen peroxide and 10 μg/ml of Avidin for 30 min and then blocked in 10% normal horse serum prepared in Tris-buffered saline (TBS) for 30 min followed by overnight incubation with primary anti-Aβ42 (4G8) antibody. Sections were rinsed in TBS and then incubated in the appropriate biotin-conjugated secondary antibody before incubation for an hour in Vectastain Elite ABC in accordance with manufacturer’s instructions. This was followed by diaminobenzidine as per instructions by manufacturer. Counterstaining of slides was achieved with hematoxylin. Microscopy was performed with an Olympus Light microscope (Olympus, Center Valley, PA).

### Long-term ShK-223 treatment trial

Adult female 5xFAD mice aged 3 mo were treated intraperitoneally twice a week until 6 mo of age with PBS or ShK-223 (dose 100 μg/kg), *n* = 10 each and also age-matched 5 female C57BL/6 mice were included in the study. At 6 mo, neurobehavioral testing including Fear conditioning and Morris Water Maze was performed. The mice were euthanized, brains isolated and the brains were utilized for immunohistochemistry studies.

### Transient transfection of HEK293 cells

HEK293 cells were maintained in DMEM with 10% FBS. Transient transfections with either pRC/CMV vector or pRC/CMV-mKv1.3 (a kind gift from Dr. Heike Wulff, UC Davis) or sham were performed for 24 h using 1 μg of plasmid DNA per well. Experiments were performed in biological triplicates.

### Aβ quantitation in paraffin-embedded mouse brains

Images from slides labeled for Aβ42 from the 6 mo 5xFAD mice following their 3 mo intraperitoneal treatment with PBS or ShK-223 were used for ImageJ analysis to determine Aβ42 plaque load and size. The images of the hippocampal region, cortex and diencephalon underwent background subtraction, then color deconvolution based on hematoxylin/DAB stains and then conversion to binary images, followed by a blinded measurement of Aβ density per region of interest using ImageJ. Plaque size as square pixels was also measured using a defined minimum threshold of 200 square pixels which excluded Aβ labeling outside of plaques.

### Reverse transcriptase quantitative PCR

CNS immune cells isolated from age-matched C57BL6 mice treated with four once daily intraperitoneal doses of sterile PBS, ShK-223 (100 μg/kg), LPS (20 μg/dose) or co-administered LPS + ShK-223 (*n* = 3/group) were washed in PBS containing RNAase inhibitors and used for RT-qPCR using our published protocols [26]. For total RNA extraction from cells, 1 mL Trizol was added to the cells, the pellets were homogenized in the Trizol by repetitive pipetting and incubated for at least 1 h at room temperature. Then, 0.2 mL of chloroform per 1 mL of Trizol was added and incubated for 2–3 min followed by centrifugation at 12000 x g for 15 min at 4 °C to obtain an upper colorless aqueous phase, an interphase and a lower red phenol chloroform phase. RNA in the aqueous phase was transferred to other tubes. 0.5 mL of 100% iso-propranolol was added to the aqueous phase per 1 mL of Trizol used and the mixture was incubated for 10 min and centrifuged at 12000 x g for 10 min at 4 °C. The pellet was washed with 1 mL of 75% ethanol per 1 mL of Trizol used (initial homogenization) and the RNA pellet was air dried and reconstituted in RNase-free water followed by incubation in a heat block set at 55–60 °C for 10–15 min. RNA concentrations were then determined via Nanodrop. RNA was reverse transcribed to cDNA using a high capacity cDNA reverse transcription kit (Ambion). Real time PCR was performed on a 7500 Fast RT-PCR instrument (Applied Biosystems) using cDNA, TaqMan PCR Master Mix (Applied Biosystems), and gene-specific TaqMan probes (Applied Biosystems) against Kcnj2 (Mm00434616_m1), Nceh1 (Mm00626772_m1), Timp2 (Mm00441825_m1), Il1B (Mm00434228_m1), Ptgs2 (Mm00478374_m1), Tmem119 (Mm00525305_m1), and Hprt (Mm03024075_m1) which was used as the ‘housekeeping gene.’ For each RNA sample, each primer set was run in triplicate. Gene expression was normalized to the internal control HPRT for primary microglia and relative expression calculated for each gene using the 2ΔΔC_T_ method after normalizing to the control [36].

### Identification of transcriptional regulators and connectivity map analysis for potential perturbagens

Connectivity map is an online library of cellular signatures from various human cell types that catalogs transcriptional responses to chemical, genetic, and disease perturbation (perturbagens) which can be used to probe relationships between diseases, cell physiology, and therapeutics (https://clue.io/about#cmap) [37]. We used this approach to identify perturbagens likely to result in a desired transcriptional profile. The perturbagens most likely to reproduce the desired transcriptional effect are identified using a summary score (range − 100 to + 100).

### Human BLSA brain samples and fisher exact test analyses for module overlap

The patient and pathological characteristics of the 47 post-mortem BLSA samples used for proteomics and the methods for tandem-mass-tag quantitative proteomics, subsequent analyses and WGCNA are available online (https://www.synapse.org/#!Synapse:syn11209141) and will be published subsequently. WGCNA identified 50 protein in this study and the correlations between module expression and neuropathological traits (BRAAK and CERAD) were investigated. A hypergeometric two-tailed Fisher’s exact test was used to determine significant overlap between the mouse microglial transcriptomic modules and BLSA proteome modules [34].

### Other statistical considerations

Graphpad Prism (Ver. 5), Cytoscape (Ver. 3.5.1) and SPSS (Ver. 22) were used to create graphs and perform statistical analyses. All data are shown as mean ± SEM. For experiments with > 2 groups, one way ANOVA was performed to detect differences across groups and post-hoc pairwise comparisons were performed using Tukey’s HSD test. Statistical significance was set at *p* value ≤0.05 for all experiments unless specified separately.

## Results

### Co-expression network analysis of microglial gene expression reveals a landscape of diverse and functionally distinct microglial activation states in AD mouse models

**Fig. 1:**
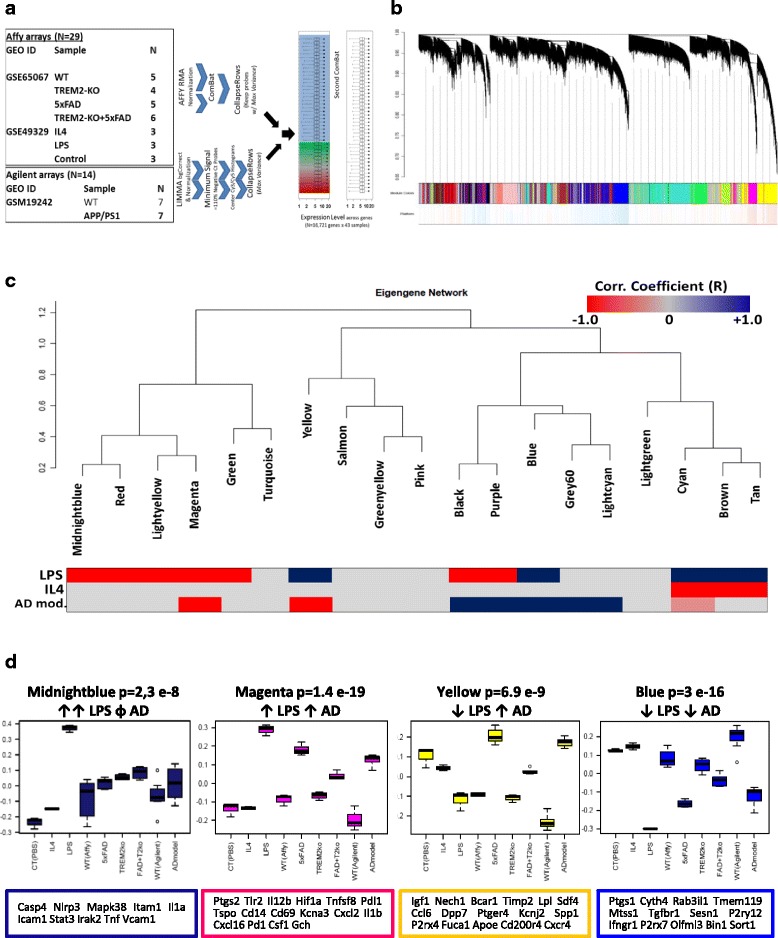
WGCNA of mouse microglial transcriptome identifies co-expression modules with distinct AD pathology-associated inflammatory profiles. **a** Three transcriptomic microarray datasets containing 43 arrays derived from CD11b^+^ mouse microglia were normalized, batch-corrected and used in a WGCNA meta-analysis. Datasets included microglia polarized in-vitro to M1-like (LPS) or M2-like (IL4) states, acutely isolated microglia from WT, 5xFAD, Trem2−/− and 5xFAD/Trem2−/− mice, and from WT and APP/PS1 transgenic mice. **b** WGCNA identified 19 distinct modules (networks) of highly co-expressed genes (See Additional file 1: Table S1). **c** Cluster dendrogram showing the 19 microglial modules and trait correlations with inflammatory status (LPS or IL4) and AD pathology (5xFAD or APP/PS1 vs. wild-type). **d** Comparison of expression of module eigengene for 4 modules of interest, across treatment conditions/traits (Hub gene members are shown in descending order of module membership or k_ME_)

**Fig. 2:**
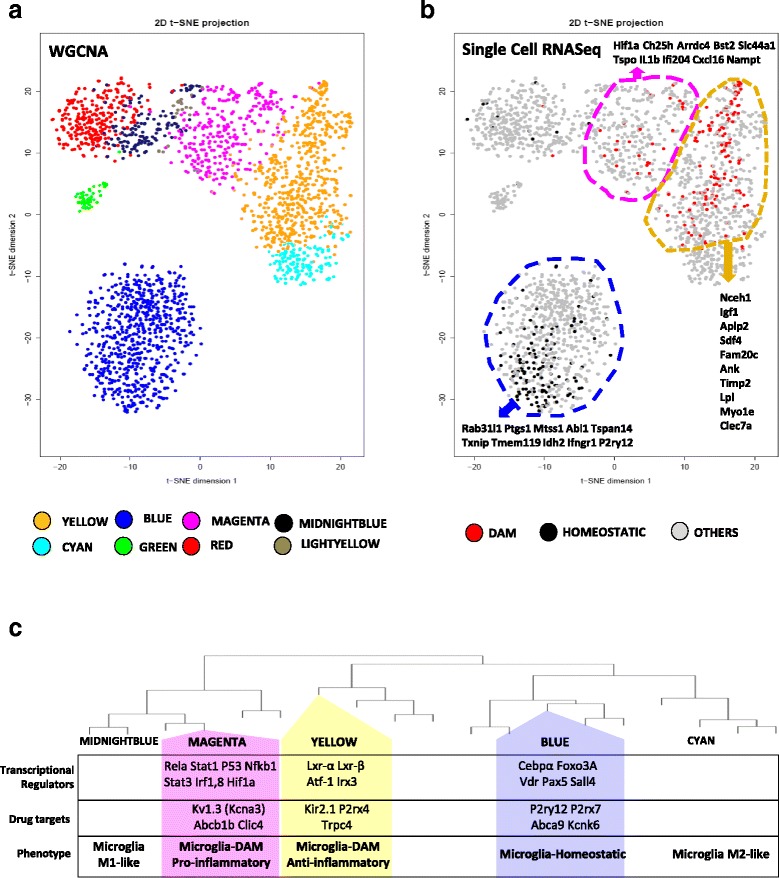
Microglial co-expression modules recapitulate homeostatic and DAM profiles and predict heterogeneity within DAM. **a** 2D-representation of trait-associated WGCNA modules using viSNE. Space between two clusters of genes is an arbitrary representation of dissimilarity or anti-correlation between expression patterns. **b** Mapping of DAM and homeostatic signature genes to WGCNA modules shows clustering of DAM genes to Yellow and Magenta modules and of Homeostatic genes to the Blue module. Genes shown in Grey represent those that were either not classified as DAM or homeostatic or were not identified by single cell RNAseq [9]. **c** Key transcriptional regulators, membrane-associated drug targets and predicted functional phenotypes of Blue, Magenta and Yellow AD-associated microglial modules (also see Additional file 7)

Data from 43 existing GEO microarray transcriptomes of CD11b^+^ microglia including primary mouse microglia treated in-vitro with M1-like activating stimulus LPS (M1-like) or M2-like stimulus IL4, and from WT, TREM2^−/−^, 5xFAD, TREM2^−/−^/5xFAD and APP/PS1 mice were batch-corrected and normalized to adjust for differences in microarray platforms (Fig. 1a) [15, 22, 23]. WGCNA of normalized expression data (16,721 genes) identified 19 modules of highly co-expressed genes (Fig. 1b-c, Additional file 8: Figure S1, Additional file 1: Table S1) including modules significantly upregulated following in-vitro LPS or IL4 treatment, representing the M1-M2 paradigm of in-vitro activation (Fig. 1c). We also identified two modules that were upregulated (Magenta and Yellow) and one that was down-regulated (Blue) across AD models (Fig. 1c-d). Within these three AD modules, we observed enrichment of pro-inflammatory genes in the Magenta module (Ptgs2, Il12b, Tlr2, Hif1a, Cd69, Il1b, Cxcl16 and Irg1) and anti-inflammatory and phagocytic genes in the Yellow module (Igf1, Dpp7, Apoe, Spp1, Cd200r4 and Lpl) [38] while the Blue module contained several homeostatic microglial genes (Tmem119, P2ry12, Mtss1 and Tgfbr1, Fig. 2d) [39].

Gene ontology (GO) analyses of key module members (Additional file 8: Figure S2, Additional file 2: Table S2) revealed that the Blue module was enriched for cytoplasmic proteins governing homeostatic functions (macromolecule biosynthesis and cellular metabolic processes). The Yellow module was enriched for cytoplasmic proteins involved in carbohydrate and lipid metabolism, cholesterol efflux, antigen presentation, oxido-reductase activity and senescence/autophagy and in contrast, the Magenta module was enriched for pro-inflammatory functions including immune signaling, cell proliferation, adhesion, antigen presentation, calcium influx and cytokine production. Pathway analyses of key members of AD modules identified transcriptional regulators (Additional file 8: Figure S3, Additional file 3: Table S3) including RelA, Stat1, p53 and Nfkb1 for Magenta, Lxrα/β and Atf-1 for Yellow and Cebpα and Foxo3a for Blue modules. These observations agree with known anti-inflammatory and Aβ-clearing effects of LXRα/β agonists in AD models [40–42] as well as the role of Cebpα in regulating PU.1 which maintains microglial quiescence [43]. These patterns of module expression across various traits and their enriched ontologies and transcriptional regulators suggest that the Magenta module is an AD-associated pro-inflammatory module, the Yellow module is an AD-associated anti-inflammatory module/pro-phagocytic and the Blue module is a homeostatic microglial module while other M1/M2-like modules are not relevant to AD pathology.

We also applied a modification of t-distributed stochastic neighbor embedding (viSNE) to normalized expression data to represent all genes in 2 or 3 dimensional space allowing for better appreciation of gene and gene cluster inter-relatedness (Fig. 2a) [35]. By layering WGCNA modules onto viSNE results, we confirmed strong agreement between viSNE clusters and WGCNA modules and observed that both pro-inflammatory (Magenta) and anti-inflammatory (Yellow) modules are closely related but are very distinct from the homeostatic (Blue) module. By representing viSNE results in 3D (Additional file 8: Figure S4, Additional file 9), we observed that homeostatic microglia may reach AD-associated states (Magenta or Yellow modules) via at least 3 distinct pathways (Additional file 8: Figure S4, Additional file 9) including a pro-inflammatory pathway (lower band) comprising of LPS-upregulated modules, a second potentially anti-inflammatory pathway (middle band) containing LPS-downregulated/IL4-upregualted modules and a third possible pathway of microglial activation (upper band). These findings support the application of network-based analyses of population-level transcriptomic data to delineate distinct microglial phenotypes, providing a framework demonstrating relatedness and interconnectivity of microglial sub-profiles.

**Fig. 3:**
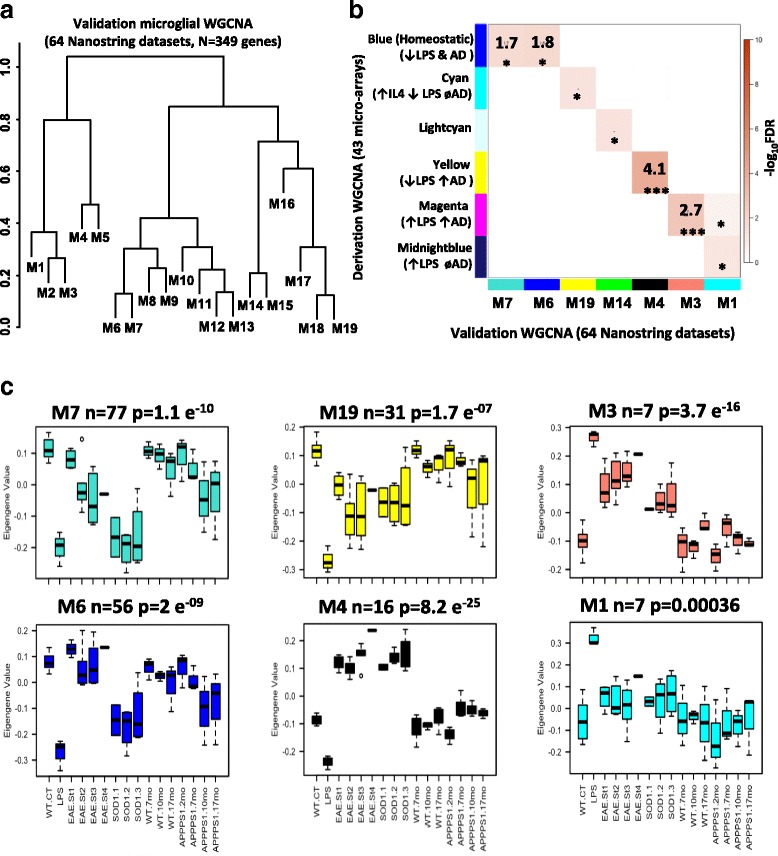
Replication co-expression analysis of microglial transcriptomic datasets across neuroinflammatory and neurodegenerative disease models. **a** Cluster dendrogram summarizing results of validation WGCNA performed using 64 existing Nanostring purified microglial transcriptomic datasets (369 genes in common across all conditions). Datasets included aging WT and APP/PS1 models (age range 2–17 mo, *n* = 24), WT and SOD1-G93A transgenic mice (*n* = 13), control and various stages of EAE in mice (*n* = 21) and WT and intra-cerebral LPS-treated WT mice (*n* = 6). Expression data were first batch-corrected followed by WGCNA to identify modules of highly co-expressed genes. **b** Module over-representation analysis (ORA) of derivation (43 microglial arrays, y-axis) and validation WGCNA (64 microglial Nanostring transcriptomic datasets, x-axis) studies. Module overlap was assessed by Fisher-exact test and the negative log_10_ transformed false discovery rate (FDR) for overlap is indicated by color intensity. Validation modules are numbered (M1–19) while derivation modules are indicated by the original color scheme. **c** Module eigengene expression and trajectories of change in module expression across various traits in the Nanostring co-expression dataset. Validation modules highlighted here were chosen based on strength of overlap from the over-representation analysis. M6 and M7 are representative of the Blue homeostatic derivation module. M4 is representative of the Yellow anti-inflammatory derivation module while M3 is representative of the Magenta pro-inflammatory derivation module. M1 is representative of LPS-induced Midnightblue derivation module while M19 is representative of the IL4-upregulated Cyan derivation module

To determine the validity of our results, we performed an external validation of WGCNA using existing microglial transcriptomic studies (Nanostring, *n* = 64 samples, 369 common genes per sample) of purified microglia isolated from WT and aging mice (2 mo–17 mo) as well as from mouse models of neuroinflammation (LPS treatment, experimental autoimmune encephalomyelitis [EAE]) and neurodegeneration (APP/PS1 model of AD pathology and SOD1-G39A transgenic model of motor neuron disease) [16]. We obtained 19 modules in this analysis and an assessment of module overlap between derivation and validation network analyses using over-representation analysis (ORA) identified modules in the validation network that were equated to Blue, Magenta and Yellow modules identified in the derivation network (Fig. 3b). Specifically, validation modules M6 and M7 represented the derivation Blue module (common hub genes Cx3cr1, P2ry12, Sall1, Csf1r and Tmem119), validation module M3 represented the derivation Magenta module (common hub gene Tlr2 and Cxcl16) and validation module M4 represented the derivation Yellow module (common hub genes Apoe, Spp1, Axl). The discernment of these validation modules across various traits in an independent expression dataset were consistent with observations in the derivation study (Fig. 3c), confirming the overall validity and generalizability of our WGCNA findings.

### Microglial modules upregulated in AD represent distinct pro-inflammatory and anti-inflammatory phenotypes within DAM

Differences in gene co-expression may indicate common upstream regulation, functional relatedness and/or restriction to same cell type or cellular compartment [34, 44]. We hypothesized that the three AD modules (Magenta, Yellow, Blue) represent transcriptomic signatures of distinct microglial sub-populations. Since single-cell RNAseq of microglia from AD mouse models has confirmed the presence of homeostatic and DAM populations [9], we mapped known DAM and homeostatic signature genes to modules identified by WGCNA (Fig. 2b, Additional file 8: Figure S4) and found that DAM genes were highly represented in both pro-inflammatory (Magenta) and anti-inflammatory (Yellow) AD modules, whereas homeostatic genes mapped to Blue and Purple modules (Fig. 2b, Additional file 8: Figure S4, Additional file 10). These observations confirm that modules identified by WGCNA using cell population data, represent homeostatic and DAM sub-populations previously identified by single-cell RNAseq and that other non-AD modules also represent distinct microglial activation states. Remarkably, we resolved DAM into two related yet functionally divergent profiles of which the Magenta module represents a novel pro-inflammatory subset.

Since both DAM modules were closely related (Fig. 2b), we hypothesized that bottleneck genes present at the Yellow/Magenta inter-modular interface with dual module membership may exist. Accordingly, we identified 35 bottleneck genes (Additional file 8: Figure S5) which mapped to this interface and had strong membership (k_ME_ ≥ 0.75) in both modules. Further characterization of these inter-modular bottlenecks could identify immune checkpoints that determine phenotypic switching between pro- and anti-inflammatory profiles. Trem2, a known immune checkpoint that regulates the transition of homeostatic microglia to DAM [9], had moderate-level membership (k_ME_ 0.45) to a module closely related to the anti-inflammatory Yellow module while the binding partner of Trem2, Tyrobp (Dap12), was a hub gene in the Yellow module [15, 17]. Since Trem2 regulates microglial survival, proliferation, effector functions [6, 15, 17] and a checkpoint in the origin of DAM in AD [9], we asked whether distinct DAM profiles emerge upstream or downstream of the Trem2 checkpoint. Our WGCNA results showed that Trem2 deletion in 5xFAD mice resulted in downregulation of both pro-inflammatory and anti-inflammatory DAM modules while upregulating the homeostatic module (Fig. 1d and Additional file 8: Figure S6) suggesting that both pro-inflammatory (Magenta) and anti-inflammatory (Yellow) modules co-emerge downstream of Trem2 but represent related yet functionally divergent pro- and anti-inflammatory profiles within DAM.

### Confirmation of pro-inflammatory and anti-inflammatory DAM profiles in AD mouse models

**Fig. 4:**
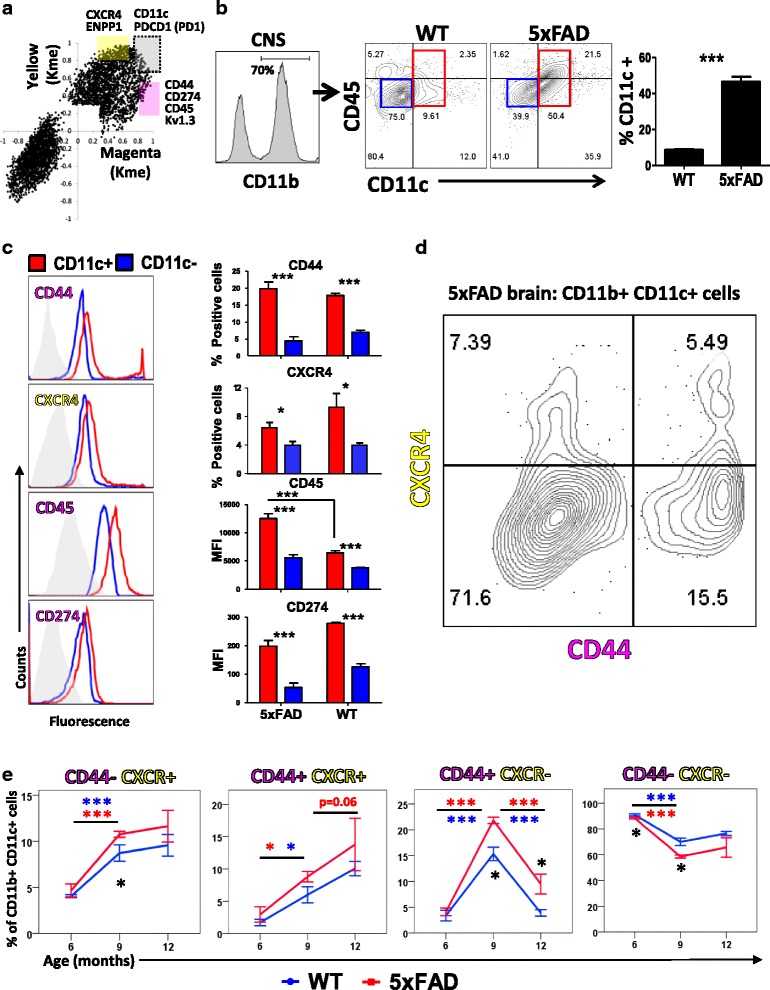
Flow cytometric phenotyping of microglia using pro- and anti-inflammatory DAM markers. **a** Selection of potential flow cytometric markers for Magenta (pro-inflammatory) and Yellow (anti-inflammatory) DAM modules in acutely isolated WT and 5xFAD microglia. Criteria included k_ME_ ≥ 0.75 for one module, ≤0.5 for the other module and predominant cell membrane localization. Yellow module: Cxcr4 (CD184); DAM-phenotype: CD11c and Pdcd11 (PD1); Magenta module: CD44, CD274 (PDL1), CD45 and Kv1.3. Cell surface functional Kv1.3 channel expression was detected by ShK-F6CA, a fluorescein-conjugated Kv1.3 channel blocker that selectively binds to Kv1.3 channels. **b** Acutely isolated CNS immune cells were gated for live cells (FSC/SSC) and then for single cells (using FSC-A/FSC-H) followed by CD11b positivity. CD11c was used as a marker of DAM based on prior RNAseq studies. CD11c^+^ DAM were gated as shown and proportions of CD11c^+^ DAM in CD11b^+^ CNS immune cells were compared between WT and 5xFAD mice (n = 6 mice/group, age 6–8 mo). **c** Comparison of pro-inflammatory DAM (CD44, CD45 and CD274) and anti-inflammatory DAM (CXCR4) specific markers within CD11c^+^ DAM in 5xFAD and WT mice (n = 6/group, age 6–8 mo). Grey histogram represents isotype control. **d** Flow cytometric phenotyping of CD11b^+^ cells in mouse brain based on CD44 and CXCR4 expression. **e** Changes in proportions of DAM subsets in aging WT and in aging 5xFAD mouse brains (*n* = 4 mice/group/time point). Pairwise and group-wise comparisons and significant differences are highlighted. Levels of significance: *p < 0.05, **p < 0.01, ***p < 0.005. Colored markers indicate within group comparisons (Red: 5xFAD, Blue: WT) while Black markers indicate inter-group differences (WT vs. 5xFAD)

To confirm the presence of distinct pro-inflammatory and anti-inflammatory DAM subsets resolved by co-expression analyses, we performed flow cytometric studies of acutely isolated CNS immune cells from adult WT and 5xFAD mice to determine whether surface markers exclusive to pro-inflammatory and anti-inflammatory DAM modules distinguish distinct sub-populations within DAM [9]. Genes with known cell surface expression and module specificity (k_ME_ ≥ 0.75 for one and ≤ 0.5 for the other, Fig. 4a) included CXCR4 (anti-inflammatory/Yellow module); and CD44, CD274, CD45 and Kv1.3 (pro-inflammatory/Magenta module). CD11c (*Itgax*) was chosen as a general marker of DAM based on existing single-cell RNAseq and confirmatory studies in human brain [9, 45].

In 6–8 mo old 5xFAD mice, CD11c^+^ DAM were more abundant in 5xFAD (≈50%) as compared to WT (< 10%) CD11b^+^ CNS immune cells, Fig. 4b). Pro-inflammatory DAM markers (CD44, CD45, CD274) and the anti-inflammatory DAM marker (CXCR4) were also increased in CD11c^+^ DAM in 5xFAD mice (Fig. 4c). While DAM from 5xFAD and WT mice had similar CD44, CD274 and CXCR4 surface expression, CD45 was much higher in DAM from 5xFAD mice. In 5xFAD CNS CD11b^+^CD11c^+^ DAM, we confirmed the presence of exclusively CD44^+^, exclusively CXCR4^+^, CD44^+^CXCR4^+^ double positive as well as CD44^−^CXCR4^−^ populations (Fig. 4d). These DAM subsets were then further studied in WT and 5xFAD mice across various age groups (6–12 mo). Aging resulted in increased proportions of CD44^−^CXCR4^+^ and CD44^+^CXCR4^+^ DAM while CD44^+^CXCR4^−^ DAM increased significantly at 9 mo of age and decreased by 12 mo of age (Fig. 4e). Double-negative CD44^−^CXCR4^−^ showed a modest but significant age-associated decline in both WT and 5xFAD mice. While the trajectories of change in all DAM subsets were similar in WT and 5xFAD mice, all age-related changes were augmented in 5xFAD DAM (Fig. 4e). Majority of DAM did not express high levels of either CD44 or CXCR4 suggesting that any additional complexity within DAM may not have been revealed by our flow cytometry panel. Recent profiling of microglia by mass cytometry [46, 47] identified several subsets of resident myeloid cells in the naive mouse brain including CD14^high^, CXCR4^high^, CCR5^high^, CD115 (Csf1r)^high^ as well as MHC-II^high^CD44^high^ subsets [47], although proportions of these subsets in AD models is unknown. Interestingly, several of these markers were hub genes of homeostatic microglia (Cx3cr1, CD115/Csf1r, CCR5), pro-inflammatory DAM (CD14, CD44) and anti-inflammatory DAM (CXCR4) modules in our WGCNA. Collectively, our results confirm distinct subsets within DAM in accordance with network-based predictions and highlight previously unappreciated phenotypic diversity within microglia. Distinct age-dependent trajectories of pro- and anti-inflammatory DAM also suggest that aging influences the balance between DAM activation states, linking aging with other determinants of AD pathogenesis.

### Identification of drug targets for inhibiting pro-inflammatory DAM and augmenting anti-inflammatory DAM profiles

**Fig. 5:**
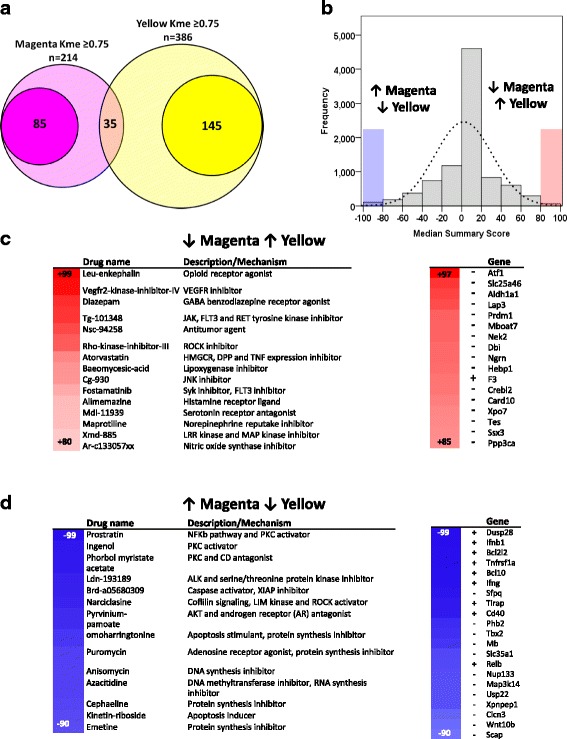
CMAP analysis pro-inflammatory and anti-inflammatory DAM genes reveals genetic and chemical perturbagens as selective modulators. **a** Venn diagram representing exclusive members and dual members of Magenta and Yellow microglial modules. **b** Distribution of perturbagens from connectivity map (CMAP) analysis based on a median summary score (range − 100 to + 100) that represents likelihood of the perturbagen resulting in the desired transcriptional profile. **c**, **d** For these analyses, the desired transcriptional profile was upregulation of anti-inflammatory and down-regulation of pro-inflammatory module expression. A summary score ≥ + 90 typically indicates a high likelihood of achieving the desired profile (c, ↓Magenta/pro-inflammatory ↑Yellow/anti-inflammatory) while a score ≤ − 90 indicates likelihood of the opposite transcriptional profile (d, ↑Magenta/pro-inflammatory ↓Yellow/anti-inflammatory). The most significant chemical perturbagens (existing drugs) and genetic perturbagens (gene suppression or over-expression) are shown

To identify therapeutic approaches to specifically inhibit pro-inflammatory DAM and promote anti-inflammatory DAM gene expression, we performed an analysis using the connectivity map (CMAP) repository of transcriptional responses in human cells [37]. From our WGCNA results, we identified 85 pro-inflammatory DAM-specific and 145 anti-inflammatory DAM-specific genes as transcriptional signatures of each DAM module (Fig. 5a) and performed CMAP analysis to identify chemical and genetic perturbagens predicted to inhibit pro-inflammatory and augment anti-inflammatory DAM gene expression (Fig. 5b), the desired profile of an ideal neuro-immunomodulatory therapy in AD. We identified several therapeutic candidate drug classes including statins, opioid receptor agonists and inhibitors of Syk/Flt3, Vegfr, Jnk and Rock pathways (Fig. 5c). Perturbagens predicted to result in the opposite less desirable phenotype (increased pro-inflammatory and decreased anti-inflammatory DAM gene expression) were also identified (Fig. 5d), possibly representing effects of environmental exposures that increase AD risk.

We also searched homeostatic and DAM modules for genes encoding membrane-associated transporters and receptors since these are more likely to represent drug targets. We identified transmembrane receptors/transporter genes (Additional file 4: Table S4) that met at least 2 of the following criteria: (1) confirmed expression and function in microglia/macrophages, (2) existing drug modulators, and (3) relevance to neurological diseases. These included K^+^ channel Kv1.3 (Kcna3), multi-drug-resistance protein Abcb1b and chloride transporter Clic4 in the pro-inflammatory DAM module; K^+^ channel Kir2.1 (Kcnj2), purinergic channel P2rx4 and transient receptor potential channel Trpc4 in the anti-inflammatory DAM module; and purinoceptors P2ry12 and P2rx7 and two-pore K^+^ channel Kcnk6 in the homeostatic module. Our pathway analyses also identified transcriptional regulators of pro-inflammatory DAM (NFkB and RelA) and anti-inflammatory DAM (LXRα/β and Atf1). Based on these *in-silico* findings, we prioritized in-vivo target validation studies in 5xFAD models using an activator of LXRα/β to promote anti-inflammatory DAM and a selective blocker of Kv1.3 channels to inhibit pro-inflammatory DAM responses.

### Promotion of anti-inflammatory DAM and Aβ phagocytosis by LXRα/β agonists in AD mouse models

**Fig. 6:**
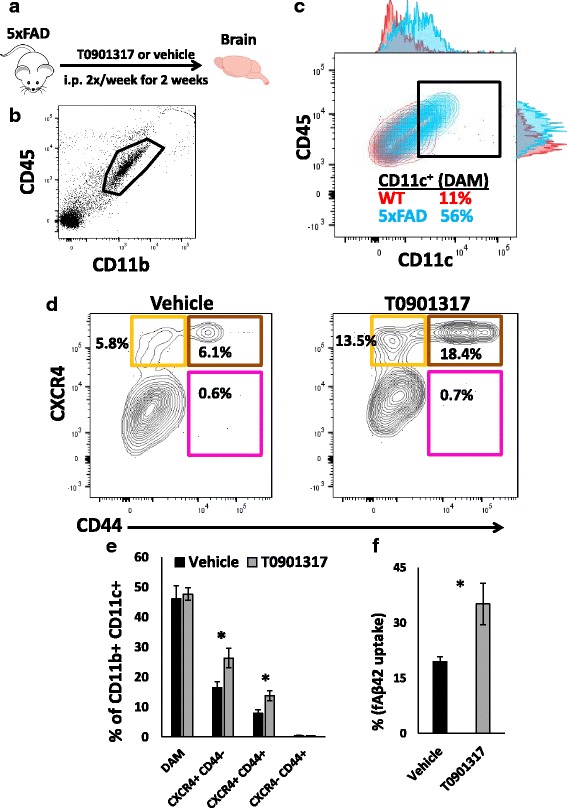
Agonists of liver-X-receptor (LXR) α/β signaling promote anti-inflammatory DAM and Aβ phagocytic activity in microglia. **a** Experimental design: 6–7 mo 5xFAD mice (females, n = 4/group) received either vehicle (20% DMSO) or a LXR α/β agonist T0901317 (30 mg/kg i.p. daily × 2 weeks) after which acutely isolated CNS immune cells were assessed for flow cytometric immuno-phenotyping as well as flow cytometric assays of fluorescent fibrillar Aβ. **b**, **c** CD11b^+^CD45^low^ CNS immune cells were assessed for CD11c expression. A comparison of CD11c expression in 5xFAD mice (blue) and WT mice (red) is shown. Gating threshold for CD11c is shown in (**c**). **d**, **e** A comparison of CD44 and CXCR4 expression profiles within CD11c^+^ DAM acutely isolated from vehicle-treated or T0901317-treated 5xFAD mice. Relative proportions of each subset (of all CD11b^+^CD45^low^CD11c^+^ DAM) are shown. Yellow represents anti-inflammatory DAM, Magenta represents pro-inflammatory DAM and Brown represents double positive cells. A quantitative analysis of various subsets of CD11c + DAM in vehicle-treated and T0901317-treated mice is shown in (**e**). **f** Comparison of ability of acutely isolated CD11b^+^CD45^low^ microglia (from vehicle-treated and T0901317-treated mice) to phagocytose fluorescent Aβ42 fibrils. *p < 0.05, **p < 0.01, ***p < 0.005

Agonists of LXRα/β, predicted to specifically promote anti-inflammatory DAM gene expression in our network analysis, have anti-inflammatory and protective effects in AD models [40, 41]. Therefore, we hypothesized that an agonist of LXRα/β pathways should increase the proportions of anti-inflammatory DAM cells as well as promote the ability of microglia to phagocytose Aβ in the 5xFAD mouse model of AD. We treated 10–12 mo old 5xFAD mice with the LXR agonist T0901317 (or vehicle) using a twice-weekly i.p. dosing regimen for 2 weeks [40, 41] (Fig. 6a-c), after which acutely isolated CD11b^+^ CNS immune cells were immunophenotyped. This late time point of disease in 5xFAD mice was chosen since majority of microglia adopt the DAM profile [9]. Treatment with T0901317 did not impact total numbers of CD11b^+^ CNS immune cells, proportions of CD45^high^ CNS-infiltrating immune cells, proportions of CD11c^+^ DAM in the brain, or peripheral CD11b^+^CD45^+^ splenocytes (data not shown). However, within DAM, we observed nearly two-fold increase in proportions of CXCR4^+^CD44^−^ (anti-inflammatory DAM) as well as CXCR4^+^CD44^+^ cells, while CXCR4^−^CD44^+^ (pro-inflammatory DAM) cells were unaltered (Fig. 6d-e).

To further determine whether augmentation of anti-inflammatory DAM is associated with increased phagocytic properties, we performed ex-vivo phagocytosis assays using acutely isolated CNS immune cells (from vehicle-treated or T0901317-treated mice) which were incubated with fluorophore-conjugated fibrillar Aβ42 followed by flow-cytometric measurement of Aβ uptake. As compared to vehicle-treated microglia, T0901317 treated mouse microglia demonstrated higher fibrillar Aβ42 phagocytosis (Fig. 6f). In summary, we observed specific augmentation of anti-inflammatory DAM as well as overall augmentation of Aβ phagocytosis by an agonist of LXRα/β, consistent with our network-based predictions and with known Aβ-lowering effects of LXRα/β agonists in AD models [42].

### Validation of Kv1.3 channels as specific regulators of pro-inflammatory DAM responses and neuropathology in AD mouse models

We also identified Kv1.3 channels as ideal therapeutic targets to inhibit pro-inflammatory DAM and possibly promote anti-inflammatory DAM responses [26, 48, 49]. Kv1.3 channels regulate effector functions of pro-inflammatory activated microglia [26, 50–52] and are highly expressed by microglia surrounding Aβ plaques in AD [48]. Kv1.3 channel blockade by the small molecule Pap1 was also found to mitigate Aβ deposition and improve neurobehavioral measures in AD mouse models [49]. However, it is unclear whether Kv1.3 channels are indeed specifically expressed by pro-inflammatory DAM in AD. Furthermore, the only class of Kv1.3 channel blockers that has successfully completed phase Ib studies in humans for psoriasis (ShK analogs) has not been tested in pre-clinical models of AD pathology [53, 54]. To address these knowledge gaps regarding Kv1.3 as a therapeutic target in AD, we performed flow cytometric studies to determine the pattern of Kv1.3 channel expression in 5xFAD and aging mice and tested the in-vivo efficacy of the ShK analog ShK-223 in acute neuroinflammatory and AD mouse models. ShK-223 was selected due to its improved stability and > 10,000-fold selectivity for Kv1.3 channels over neuronal Kv channels [53, 54].

**Fig. 7:**
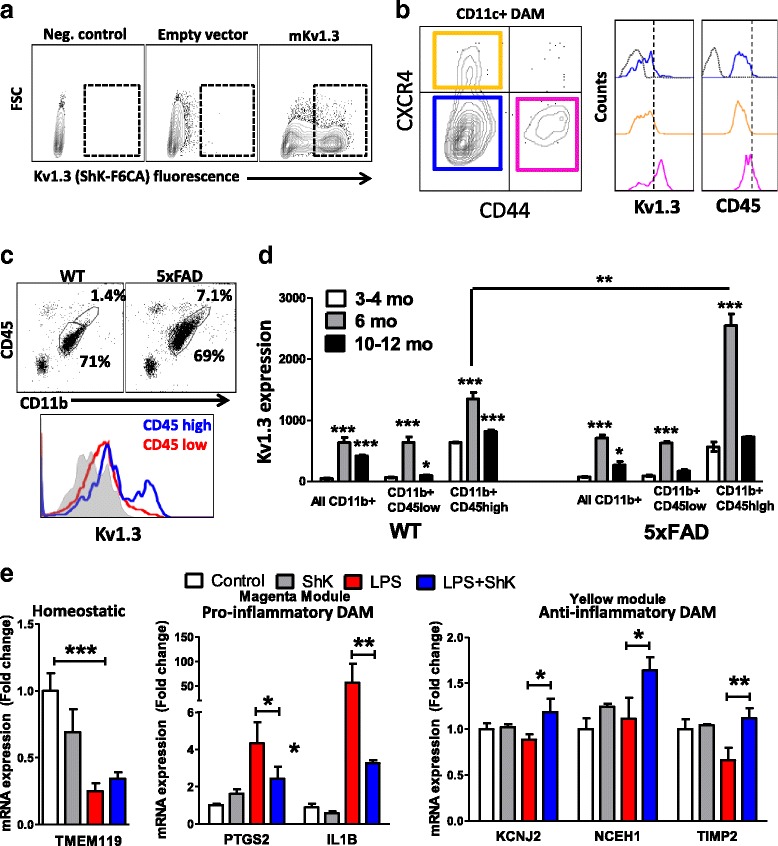
Kv1.3 channels are expressed by pro-inflammatory DAM and regulate pro-inflammatory DAM genes. **a** Confirmation of specificity of flow cytometric assay of functional Kv1.3 channels: HEK293 cells were transiently transfected with 1 μg of pRC/CMV (empty vector) or pRC/CMV-mKv1.3 (mouse Kv1.3) after which ShK-F6CA was added (final concentration 100 nM) × 30 min, followed by flow cytometry (3 independent experiments were performed). Electrophysiological confirmation of Kv1.3 current expression was performed by whole-cell patch clamp (data not shown). **b** Expression of Magenta module markers Kv1.3 and CD45 in subsets of CD11c^+^ DAM in 5xFAD mice (Homeostatic: CD44^−^CXCR4^−^, Magenta/pro-inflammatory-DAM: CD44^+^CXCR4^−^, Yellow/anti-inflammatory-DAM: CXCR4^+^CD44^−^ and double-positive CD44^+^CXCR4^+^ DAM) in adult 5xFAD mice (*n* = 5 mice, age 6–8 mo). Grey histogram represents isotype control (for CD45) or negative control (unlabeled cells for ShK-F6CA). **c** Gating of CNS immune cells based on CD11b and CD45 into CD11b^+^CD45^low^ (resident microglia) and CD11b^+^CD45^high^ subpopulations (Top). Kv1.3 surface expression in CD45^high^ and CD45^low^ subsets of CD11b^+^ cells, is shown below. **d** Comparison of Kv1.3 expression in CD45^low^ and CD45^high^ subsets of CD11b^+^ CNS immune cells in 3 age groups of WT and 5xFAD mice (*n* = 3 mice/group/time point. Post-hoc statistical tests: *p < 0.05, **p < 0.01, ***p < 0.005. **e** Comparison of gene expression in acutely isolated CNS immune cells isolated from 6 mo WT mice treated with saline, ShK-223, LPS or LPS + ShK-223 (4 daily i.p. doses, *n* = 3/group). Selected module markers: Homeostatic: *Tmem119*, Pro-inflammatory DAM: *Il1b*, *Ptgs2*; Anti-inflammatory DAM: *Kcnj2*, *Nceh1*, *Timp2*

To detect functional cell-surface Kv1.3 channels expressed on acutely isolated CNS immune cells, we used a validated flow-cytometric approach [26, 27] in which cells are incubated with a fluorescent Kv1.3 blocker ShK-F6CA which selectively binds to functional Kv1.3 channels. In HEK293 cells, we confirmed that ShK-F6CA binding was only seen in cells transfected with pcDNA3.1-Kv1.3 but not with empty vector (Fig. 7a). Within subpopulations of CD11b^+^CD45^low^ microglia, we confirmed that surface expression of Kv1.3 was higher in exclusively CD44^+^ microglia (pro-inflammatory DAM) cells as compared to homeostatic microglia (CD11b^+^CD11c^−^ or CD11c^+^CD44^−^CXCR4^−^) or exclusively CXCR4^+^ microglia (anti-inflammatory DAM) (Fig. 7b). Using the traditional approach of classifying CD11b^+^ CNS immune cells into CD45^low^ (microglia) and CD45^high^ (activated microglia or peripherally derived CNS-infiltrating macrophages) subpopulations, we found a greater proportion of CD45^high^ cells in 5xFAD mice (Fig. 7c) and as we previously reported [26], Kv1.3 channel expression was highest in CD45^high^ CNS immune cells. In 5xFAD compared to WT mice, Kv1.3 channels were strongly upregulated by CD11b^+^CD45^high^ CNS immune cells. Maximal Kv1.3 upregulation by CD45^low^ and CD45^high^ CD11b^+^ immune cells was observed at 6 mo of age, after which Kv1.3 expression decreased (Fig. 7d) a finding that is supported by published electrophysiological data [49].

Next, we performed in-vivo studies to determine whether Kv1.3 channel inhibition by ShK-223 can inhibit pro-inflammatory DAM and augment anti-inflammatory DAM gene expression in the model of acute neuroinflammation induced by low dose systemic LPS administration [26, 54]. We found that ShK-223 treatment inhibited LPS-induced upregulation of proinflammatory genes (Ptgs2, Il1b) and augmented expression of anti-inflammatory DAM genes (Kcnj2, Nceh1, Timp2) without affecting LPS-induced suppression of homeostatic gene Tmem119 (Fig. 7e). The ability of systemic ShK-223 to modulate microglial function in this model indirectly supports its CNS bioavailability.

**Fig. 8:**
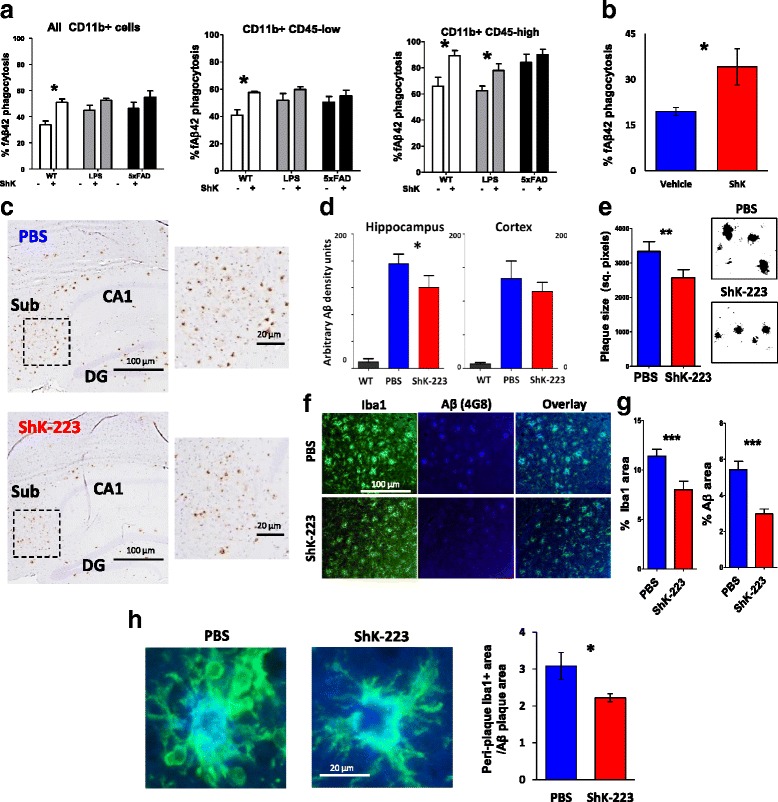
Kv1.3 channel blockade promotes Aβ phagocytosis and limits amyloid β burden in 5xFAD mice. **a** Comparison of ex-vivo phagocytosis of fluorescent Aβ42 fibrils by acutely isolated CNS CD11b^+^ cells from WT (sham-treated or LPS-treated for 4 days) and 5xFAD mice (age 6–8 mo, *n* = 4 mice/group). Cells were loaded with fluorescent fibrillar Aβ42 in the presence of either sham or ShK-223 (100 nM) for 1 h prior to labeling with anti-CD11b and anti-CD45 antibodies. **b** Comparison of ex-vivo phagocytic capacity for fluorescent Aβ42 fibrils by acutely isolated CNS CD11b^+^ cells from sham-treated or ShK-223-treated 5xFAD mice (*n* = 4/group). ShK-223 was administered twice a week (i.p., 100 μg/kg) in this study. **c-g** In this study of ShK-223 in 5xFAD mice, mice were treated with PBS or ShK-223 (100 μg/kg) between 3 and 6 mo (*n* = 10 mice/group) and brains were fixed for immunohistochemistry. **c** Comparison of hippocampal Aβ plaques in PBS-treated (top) and ShK-223-treated (bottom) mice (Right: Higher magnification image). **d** Quantification of Aβ plaque burden in the hippocampus and frontal cortex. **e** Comparison of hippocampal (subiculum) Aβ plaque size in PBS- and ShK-223-treated mice. **f**, **g**, **h** Immunofluorescence images showing Iba1 (Green) and Aβ (Blue) immunoreactivities in the hippocampus of PBS- and ShK-223-treated mice. **g** Quantitative analyses of Iba1 expression (% area) and Aβ plaque burden. **h** Quantitative analysis of peri-plaque Iba1 immunoreactivity comparing PBS- and ShK-223-treated mice. 5–8 plaques of relatively similar size were selected from each mouse and Iba1^+^ area was normalized to the plaque area and compared across groups. *p < 0.05, **p < 0.01, ***p < 0.005

To determine whether pro-inflammatory DAM inhibition by Kv1.3 blockade promotes Aβ phagocytic activity, we acutely isolated CNS immune cells from untreated and LPS-treated WT and age-matched 6 mo old 5xFAD mice and exposed them to fluorescent Aβ42 fibrils in the presence or absence of ShK-223. ShK-223 augmented phagocytic capacity in WT CD11b^+^ immune cells, and a more robust augmentation was seen in the CD45^high^ subset of CD11b^+^ cells, the same subset that also highly expresses Kv1.3 channels (Fig. 8a). No effect of ShK-223 was seen in CD11b^+^CD45^high^ cells in 5xFAD mice, although these already had a very high baseline level of phagocytic activity suggesting saturation. In a separate experiment, 5xFAD mice (age 6 months) received i.p. ShK-223 for 30 days, after which acutely isolated CD11b^+^ CNS immune cells were assessed for Aβ phagocytosis. We observed that ShK-223 treatment resulted in an overall augmentation of Aβ phagocytosis (Fig. 8b). Despite effective uptake, Aβ clearance by microglia in AD may be ineffective despite phagocytic uptake [55]. Therefore, we tested the effect of Kv1.3 blockade on fAβ42 compartmentalization into mature Lamp1-positive phago-lysosomes. BV2 microglia were loaded with fluorescent Aβ fibrils, subsequently washed and cultured with or without ShK-223 for 72 h. ShK-223 significantly increased Lamp1 and Aβ co-localization at 16 and 24 h time points (Additional file 8: Figure S7).

To determine whether long-term Kv1.3 channel blockade by ShK-223 results in lower burden of Aβ in 5xFAD mice, we initiated intra-peritoneal treatments with ShK-223 at 3 mo of age and continued therapy until 6 mo when 5xFAD mice, in our experience, show robust Aβ deposition and neuroinflammation without neuronal loss or neurobehavioral deficits. This time point was chosen as it represents an optimal therapeutic window for neuro-immunomodulation in humans with AD pathology. Following 3 mo of ShK-223 therapy, we observed significantly lower Aβ plaque burden (Fig. 8c-d) in the hippocampus, smaller Aβ plaque size (Fig. 8e) compared to sham-treated mice as well as decreased Iba1-immunoreactivity around Aβ plaques (Fig. 8f-g). To determine whether this observed decrease in microgliosis was a result of ShK-223’s effect of lower Aβ burden rather than a direct effect on microglial activation, we assessed Iba1 immunoreactivity in peri-plaque microglia and found that ShK-223 treatment was associated with lower Iba1 positivity around Aβ plaques even after accounting for differences in plaque size (Fig. 8h). Overall, these results show that Kv1.3 blockade can inhibit pro-inflammatory DAM gene expression and promote anti-inflammatory DAM gene expression while promoting phagocytic Aβ clearance, a protective function of DAM in AD.

### Genetic and pathological links of microglia subtypes in human AD brain

**Fig. 9:**
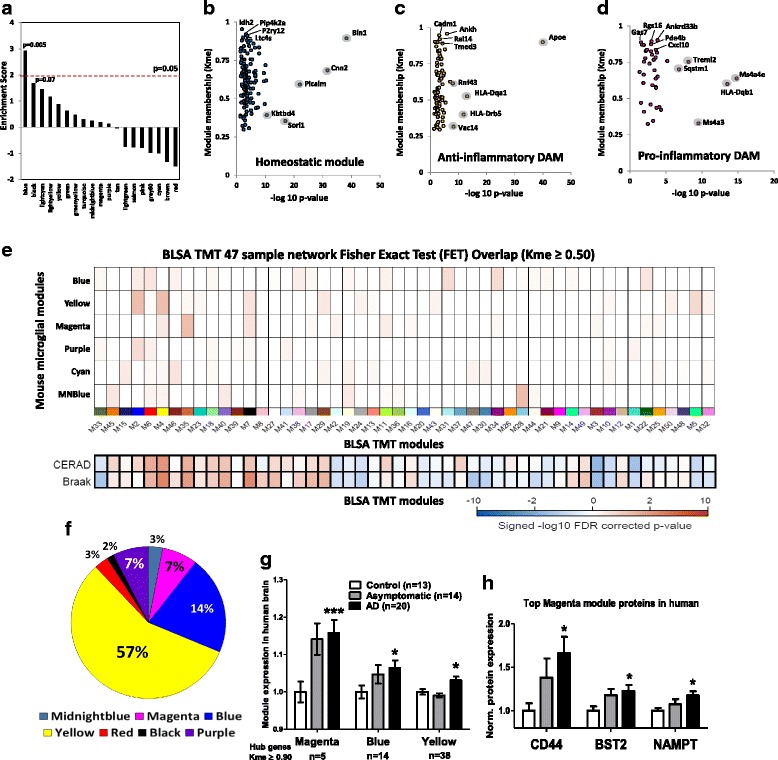
Identification of AD-risk genes as key members of homeostatic, pro-inflammatory DAM and anti-inflammatory DAM modules. **a** Enrichment analysis of GWAS-identified AD risk genes in mouse microglial modules (1234 genes identified by MAGMA were used for this analysis [34]). An enrichment score of ≥1.96 (red line) corresponds to significant enrichment (p < 0.05). **b-d** AD GWAS hits specific to (**b**) Blue (homeostatic), (**c**) Yellow (anti-inflammatory DAM) and (**d**) Magenta (pro-inflammatory DAM) modules. Top 5 genes based on strength of disease association (X-axis: -log10 *p*-value) are highlighted by a gray silhouette and top 5 genes based on module membership (k_ME_, Y-axis) are shown (also see Additional file 5: Table S5, Additional file 6: Table S6). **e-h** Analyses performed using quantitative proteomic data obtained from 47 post-mortem human frontal cortices (BLSA, *n* = 20 confirmed AD cases with cognitive decline, *n* = 13 controls without any AD pathology and *n* = 14 cases with AD pathology without cognitive symptoms). **e** Overlap of mouse modules [Blue/homeostatic, Yellow/anti-inflammatory DAM, Magenta/pro-inflammatory DAM, Purple (homeostatic), Cyan (IL4-upregulated module) and Midnightblue (LPS-upregulated module)] with human protein modules by hypergeometric one-tailed Fisher-exact test. Color intensity indicates strength of significance after Benjamini-Hochberg FDR correction. Strongest FDR-corrected *p*-values (0.075) were seen for overlap between Yellow and Magenta mouse microglial modules with M2 (unadjusted *p* = 0.0013), M4 (unadjusted *p* = 0.0014) and M35 (unadjusted p = 0.0014) human proteomic modules. Y-axis: Mouse WGCNA modules; X-axis: Protein BLSA modules. Correlations between BLSA modules with CERAD and Braak neuropathological grades of AD are shown. **f** Distribution of mouse microglial module hub genes (k_ME_ ≥ 0.90) in human BLSA AD proteome. **g** Comparison of aggregate expression of Magenta, Yellow and Blue mouse microglial modules in control, asymptomatic AD and clinical AD. Modules with ≥5 hub genes (k_ME_ ≥ 0.8) were included. Median module expression was calculated and compared across groups. **h** Normalized protein expression data of top three proteins from the pro-inflammatory Magenta module (CD44, Bst2 and Nampt) are shown

To investigate the relevance of homeostatic, pro- and anti-inflammatory DAM signatures in human AD, we first determined whether the AD-associated microglial modules identified in mice were enriched for known AD genetic risk factors identified by GWAS in humans. Although GWAS of AD patients has identified causative immune gene polymorphisms in AD [34, 56, 57], the aspects of CNS immune responses regulated by AD-associated GWAS hits have not been clarified. From 1234 AD-risk associated genes identified using Multi-marker Analysis of GenoMic Annotation (MAGMA) of GWAS in late-onset AD [34, 56], we found that AD-associated GWAS hits were highly enriched in the homeostatic (Blue) module (Fig. 9a, *p* = 0.005). Genes with strong AD associations and high module memberships within the homeostatic module included Bin1, Cnn2, Picalm and Sorl1 (Fig. 9b). The selective enrichment of AD risk genes in homeostatic microglia suggests that perturbations in homeostatic microglial functions such as immune surveillance are causally implicated in late-onset AD. Although AD GWAS genes were not specifically enriched in the pro- and anti-inflammatory DAM modules, several AD-associated genes were also identified in the anti-inflammatory Yellow module (Apoe, Hla-dqa1) as well as the pro-inflammatory Magenta module (Ms4a4e, Hla-dqb1 and Treml2) (Fig. 9c-d, Additional file 5: Table S5, Additional file 6: Table S6).

Next, we determined whether homeostatic microglial and DAM proteins are expressed in human AD brain and whether their expression is associated with AD pathology. We interrogated a quantitative proteomics dataset (47 post-mortem human brains from the Baltimore Longitudinal Study of Aging) in which WGCNA identified distinct AD-associated protein modules (https://www.synapse.org/#!Synapse:syn11209141). In this dataset from 20 clinical AD, 14 asymptomatic (pre-clinical) AD and 13 non-disease controls, 5084 of 6532 gene symbols were present in our mouse microglial network. Among these, we identified genes with at least moderate membership (k_ME_ ≥ 0.50) to mouse microglial modules and then determined the overlap between mouse microglial WGCNA modules and human brain protein modules. Anti-inflammatory DAM and pro-inflammatory DAM module members overlapped (FDR < 10%) with human protein modules (M2, M4, M35, unadjusted *p*-values < 0.01) that were also positively associated with Braak and CERAD neuropathological grades in human AD (Fig. 9e) [58]. We also found 67 hub genes (Fig. 9f) of mouse microglial modules (k_ME_ ≥ 0.90) within the human brain proteome which predominantly mapped to homeostatic, pro- and anti-inflammatory DAM mouse microglial modules. Using an aggregate expression of hub genes to represent overall expression of each module in human brain, we found that upregulation of the pro-inflammatory DAM module preceded clinical diagnosis of AD while the anti-inflammatory DAM module was upregulated modestly only in cases with symptomatic AD (Fig. 9g) while the homeostatic module showed less robust increase with AD progression. Normalized expression data of the top 3 pro-inflammatory DAM hub genes (CD44, Cst2 and Nampt) identified in the human brain proteome are shown in Fig. 9h, demonstrating a gradual increase in expression with accumulating AD pathology. These genomic and proteomic validation studies suggest a causal role for early dysregulation of microglial homeostatic mechanisms in AD and confirm the expression of both pro- and anti-inflammatory DAM modules in human AD and suggest the emergence of pro-inflammatory DAM in pre-clinical and therapeutically meaningful stages of AD.

## Discussion

**Fig. 10:**
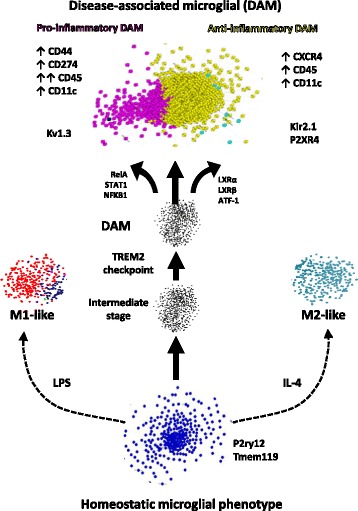
A cohesive model of homeostatic and distinct pro- and anti-inflammatory subsets of DAM in neurodegeneration. Transcriptional regulators, cell surface markers and key drug targets are shown. Each dot in this figure represents a single gene. Colors represent module membership (Blue: Homeostatic module, Red and Midnightblue: M1-like modules not associated with AD, Cyan: M2-like module not associated with AD, Black: intermediate stages, Yellow: anti-inflammatory DAM module upregulated in AD, Magenta: pro-inflammatory DAM module upregulated in AD). Upward and downward arrows indicate direction of change in gene/protein expression. Double arrow (for CD45) indicates higher degree of change in pro-inflammatory DAM as compared to anti-inflammatory DAM subsets

A comprehensive understanding of molecular and functional characteristics and regulators of microglial phenotypes in AD is critical to developing effective neuro-immunomodulatory therapies. By integrating comprehensive network analyses of microglial transcriptomic datasets with validation studies, we have developed a framework of microglial activation states in AD (Fig. 10) that allowed us to predict and confirm molecularly distinct and functionally divergent pro-inflammatory and anti-inflammatory profiles within DAM in AD mouse models, both of which emerge down-stream of the Trem2 immune checkpoint [9]. While pro-inflammatory DAM are characterized by higher expression of CD44, CD45 and Kv1.3 channels and are regulated by NFkB, Stat1 and RelA pathways, anti-inflammatory DAM are characterized by CXCR4 expression and regulated by LXRα/β. Using CD44 and CXCR4 as surface markers of pro-inflammatory and anti-inflammatory DAM respectively, flow-cytometric validation studies confirmed the existence of distinct subsets of exclusive CD44^+^ and CXCR4^+^, double-positive as well as double-negative DAM subsets, each with distinct age-dependent trajectories of change that were accentuated in the 5xFAD model of AD pathology. As predicted by our microglial transcriptional framework, we also found that promotion of anti-inflammatory DAM and inhibition of pro-inflammatory DAM were achievable via LXR agonism and Kv1.3 channel inhibition. We also found that pro-inflammatory DAM proteins are increased in human AD at pre-clinical stages and are positively associated with tau and neurofibrillary tangle pathology, emphasizing the therapeutic relevance of our findings.

By targeting decreased pro-inflammatory DAM and increased anti-inflammatory DAM as the desired effects of ideal immuno-modulatory strategies in AD, we used CMAP to identify and prioritize several classes of existing drugs that can be safely used in humans including statins, Syk inhibitors and norepinephrine reuptake inhibitors that are predicted to selectively inhibit pro-inflammatory DAM while promoting anti-inflammatory DAM. Therefore, our results provide a compelling rationale for testing these existing classes of drugs in human AD preventative trials. We also independently validated blockers of Kv1.3 channels and agonists of the LXRα/β pathway as promising therapeutics in neurodegenerative disorders [42, 49]. Kv1.3 is a voltage-gated K^+^ channel that despite low transcript level expression, is highly expressed at the channel level in immune cells where it regulates calcium signaling and effector functions [9, 10, 59]. The homo-tetrameric form of Kv1.3 channels that is susceptible to blockade by ShK analogs (such as ShK-223) is uniquely expressed in the brain by activated microglia while Kv1.3 hetero-tetramers that are resistant to ShK analogs are expressed by some cortical neurons [60, 61]. Kv1.3 channels are highly expressed by pro-inflammatory microglia in mouse models of refractory seizures as well as radiation neurotoxicity and recently, Kv1.3 blockade by Pap1 was shown to have amyloid-lowering and neuroprotective effects in AD mouse models [26, 48, 49, 51, 62, 63]. In our studies, Kv1.3 channel blockade by ShK-223 reduced the expression of pro-inflammatory DAM genes while promoting the expression of anti-inflammatory DAM genes and promoted phagocytic uptake and clearance of Aβ. Kv1.3 channels were also highly upregulated by CD11b^+^CD45^high^ CNS immune cells in the 5xFAD brain, consistent with previous findings of increased Kv1.3 expression in microglia in human AD [48]. Our pre-clinical findings using ShK-223 are of translational importance because a Kv1.3-blocking ShK analog (dalazatide or ShK-186) that is very similar to ShK-223, is the only selective Kv1.3 blocker to have successfully completed early phase human studies for systemic autoimmunity [53]. We found that systemically administered ShK-223 can modulate CNS immune responses in both acute neuroinflammatory [26] and chronic neurodegenerative disease models, suggesting its CNS bioavailability, while clearly demonstrating ability to impact neuroimmune responses. In addition to Kv1.3 channels, we also found that an agonist of LXR:RXR nuclear receptor-mediated transcriptional pathway increases the proportions of anti-inflammatory DAM and promotes Aβ phagocytosis by microglia in AD models, providing a novel mechanism for amyloid-lowering effects of LXR agonists in AD mouse models [42].

Beyond these therapeutic implications, our network-based framework of microglial gene profiles, including homeostatic, pro-inflammatory and anti-inflammatory DAM and AD-unrelated activation states, can be applied as a resource to guide target selection, experimental design and development of novel mouse models to study immune mechanisms in neurodegeneration including genetic strategies to achieve conditional microglia subtype-specific gene expression or deletion [64, 65]. For example, if a microglial gene relevant to neurodegeneration at an early stage is to be investigated, it would be most appropriate to use a conditional and cell-specific model, such as the tamoxifen-inducible Cx3cr1 Cre recombinase mouse, since Cx3cr1 is only expressed by homeostatic microglia and is downregulated by DAM [66]. On the other hand, if a pro-inflammatory DAM-specific gene is to be investigated (eg: Ptgs2/Cox2 or Tlr2), an inducible CD11c/Itgax Cre model would be more appropriate since CD11c is specifically upregulated by DAM while Cx3cr1 is downregulated [9]. Development of AD-relevant transgenic mice conditionally lacking or overexpressing pro- or anti-inflammatory DAM genes could help us further understand the roles of these functionally divergent DAM profiles at various stages of AD pathogenesis and aging.

The framework of homeostatic, pro-inflammatory DAM and anti-inflammatory DAM profiles in AD we uncovered has also allowed us to better understand immune consequences of AD risk genes including Bin1, Apoe and Treml2 [56]. Of the top AD-associated genes in the homeostatic module, Bin1 has the highest module membership suggesting a direct role for this gene in regulating homeostatic microglial functions which may be perturbed early in AD. Bin1 is a regulator of endocytosis that is associated with neurofibrillary tangle pathology in AD and is highly expressed in microglia and oligodendrocyte precursors [59]. Unique splicing of Bin1 in microglia and oligodendroglia not seen in neurons or astrocytes has also been suggested [59, 67] and Bin1 has also been identified as a highly abundant microglial protein in a recent microglial proteome [68] although the exact role of Bin1 in homeostatic microglia and in AD pathogenesis remains unclear. In the anti-inflammatory/phagocytic DAM module, Apoe was identified as a hub gene strongly associated with AD risk. This agrees with other lines of evidence confirming the role of Apoe in regulating DAM responses and Aβ-clearance in AD [16, 21]. In the pro-inflammatory DAM module, we identified Treml2 as the gene with highest module membership associated with AD risk. Treml2 has opposing roles to Trem2 in microglia [69], agreeing with the overall concept that pro-inflammatory DAM emerge down-stream of the Trem2 checkpoint after which they may diverge from anti-inflammatory DAM. In addition to these highlighted genes, our transcriptomic network identifies several other AD risk genes as potentially having novel roles in microglial activation and dysfunction in AD, providing a resource for the community and several directions for future investigations.

## Conclusion

In summary, our study highlights the value of applying unbiased co-expression analytic approaches to cell population-level transcriptomic data to unravel functionally and molecularly distinct cellular profiles that may not have been detected by single cell profiling studies [10]. By applying WGCNA to pure CD11b^+^ transcriptomic datasets, we could recapitulate the spectrum of homeostatic microglia and DAM in AD models, in addition to resolving DAM into anti-inflammatory/phagocytic and pro-inflammatory profiles, thereby providing a framework for validation and therapeutic studies. The application of co-expression network analysis to omics data from purified cell populations may allow comprehensive examination and integration of not just transcriptome, but also the proteomic and metabolomic signatures of microglial and adaptive immune responses in aging and neurodegenerative diseases.

## Additional files

Additional file 1: **Table S1.** Mouse microglial transcriptomic modules identified by Weighted Correlation Network Analysis (WGCNA). (XLSX 5372 kb)

Additional file 2: **Table S2.** Gene Ontology (GO) analyses of Blue, Magenta, Yellow and Midnightblue mouse microglial modules. (XLSX 110 kb)

Additional file 3: **Table S3.** Identification of potential transcriptional regulators of mouse microglial modules. (XLSX 13 kb)

Additional file 4: **Table S4.** Putative membrane-associated drug targets and regulators of Blue, Magenta, Yellow and Midnightblue microglial transcriptomic modules. (XLSX 12 kb)

Additional file 5: **Table S5.** Enrichment analysis of GWAS-identified human AD-risk genes in mouse microglial modules. (XLSX 22 kb)

Additional file 6: **Table S6.** Top AD risk genes in Blue, Magenta and Yellow AD-associated microglial modules. (XLSX 22 kb)

Additional file 7: **Table S7.** Changes in expression of AD-associated Blue, Magenta and Yellow microglial modules in aging WT and APP/PS1 mice. (XLSX 10 kb)

Additional file 8: **Figure S1**, related to Figure 1. Expression of microglial transcriptomic modules identified by WGCNA. **Figure S2**, related to Figure 1. Gene ontology analysis reveals distinct cellular localization and functional profiles of microglial networks in AD. **Figure S3**, related to Figure 1. Identification of transcriptional regulators of AD-associated microglial modules. **Figure S4**, related to Figure 2. A transcriptomic landscape of microglial activation states in AD. **Figure S5**, related to Figure 2. Magenta and Yellow modules likely emerge as distinct subtypes from a common microglial precursor state (Additional file 7: Table S7). **Figure S6**, related to Figure 2. Pro- and anti-inflammatory DAM networks emerge downstream of the Trem2-mediated immune checkpoint in AD. **Figure S7**. ShK-223 promotes compartmentalization of Aβ in mature phagolysosomes. (DOCX 2159 kb)

Additional file 9: 3D viSNE representation of microglial modules. Color is indicative of the respective module color. (HTML 3835 kb)

Additional file 10: 3D viSNE representation of microglial modules: DAM (Red) and Homeostatic (Black) genes identified by single-cell RNAseq mapped to WGCNA modules. (HTML 3818 kb)

## Abbreviations

AD: Alzheimer’s disease
Aβ: Amyloid beta
DAM: Disease-associated-microglia
LPS: Lipopolysaccharide
