# Supplementary material for: Identification and therapeutic modulation of a pro-inflammatory subset of disease-associated-microglia in Alzheimer’s disease
Source: Mol Neurodegener. 2018 May 21;13:24. doi: 10.1186/s13024-018-0254-8 (PMC5963076; doi:10.1186/s13024-018-0254-8)
Supplement: Supplementary file 8 — Figure S1, related to Figure 1. Expression of microglial transcriptomic modules identified by WGCNA. Figure S2, related to Figure 1. Gene ontology analysis reveals distinct cellular localization and functional profiles of microglial networks in AD. Figure S3, related to Figure 1. Identification of transcriptional regulators of AD-associated microglial modules. Figure S4, related to Figure 2. A transcriptomic landscape of microglial activation states in AD. Figure S5, related to Figure 2. Magenta and Yellow modules likely emerge as distinct subtypes from a common microglial precursor state (Additional file 7: Table S7). Figure S6, related to Figure 2. Pro- and anti-inflammatory DAM networks emerge downstream of the Trem2-mediated immune checkpoint in AD. Figure S7. ShK-223 promotes compartmentalization of Aβ in mature phagolysosomes. (DOCX 2159 kb) [file 13024_2018_254_MOESM8_ESM.docx]

**Identification and therapeutic modulation of a pro-inflammatory subset of disease-associated-microglia in Alzheimer’s disease**

Srikant Rangaraju,*^1^ Eric B. Dammer,*^1^ Syed Ali Raza,^1^ Priyadharshini Rathakrishnan,^1^ Hailian Xiao,^1^ Tianwen Gao,^1^ Duc Duong,^2^ Michael W. Pennington,^3^ James J. Lah,^1^ Nicholas T. Seyfried,^2^ Allan I. Levey^1^

*Co-first authors

^1^ Department of Neurology, Emory University, Atlanta, GA USA.

^2^ Department of Biochemistry, Emory University, Atlanta, GA USA

^3^ Peptides International, Louisville, KY USA

Corresponding author/Lead contact: Srikant Rangaraju [srikant.rangaraju@emory.edu](mailto:srikant.rangaraju@emory.edu)

Supplemental Figures: 7

Supplemental Tables (excel spreadsheets): 7

Other supplemental items (interactive html files): 2


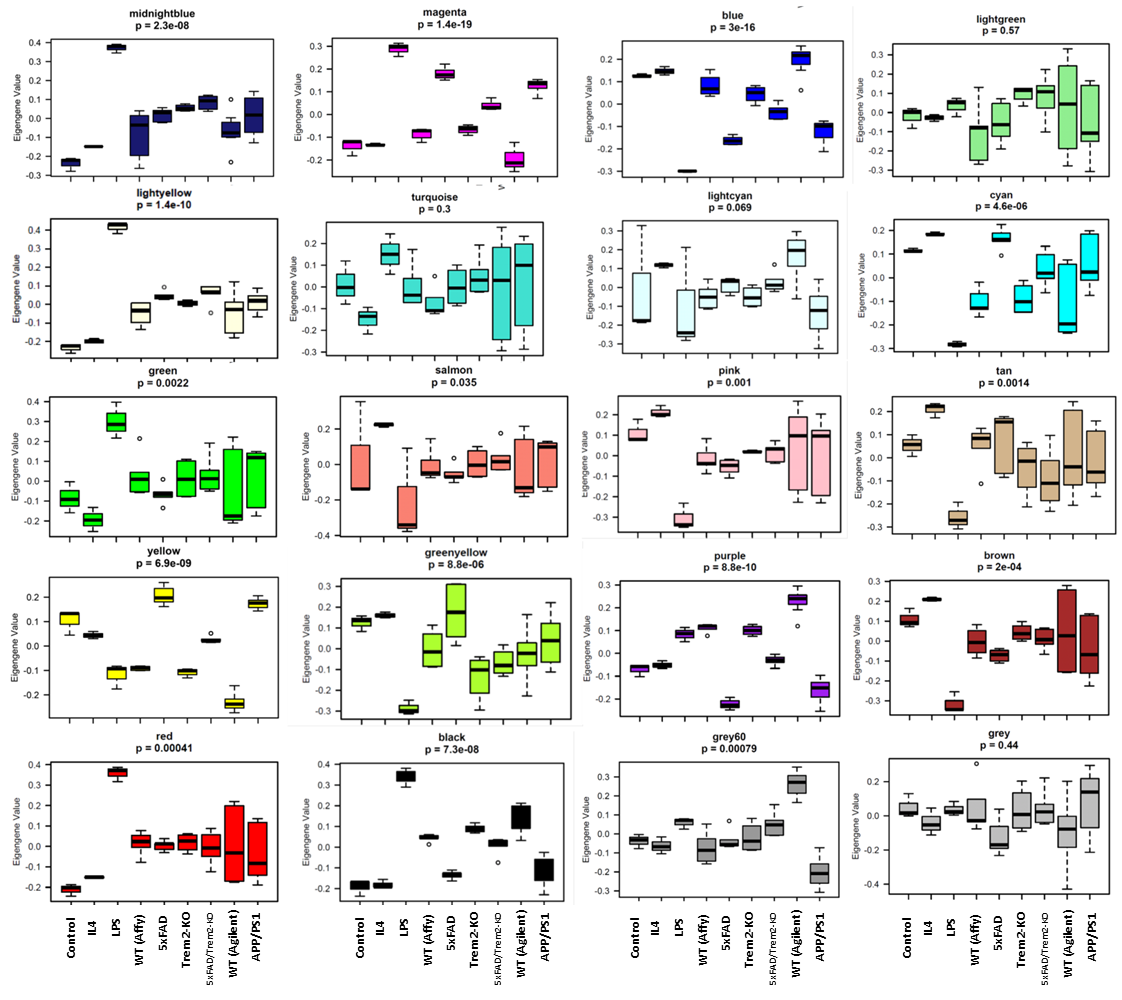


**Figure S1, Related to Figure 1.** **Expression of microglial transcriptomic modules identified by WGCNA.** Boxplots represent the eigengene expression (median and inter-quartile range) of each module across various traits in the microglial transcriptomic meta-analysis. Traits include *in-vitro* (control, IL4 and LPS) and *in-vivo* conditions (Affymetrix array: WT, 5xFAD, Trem2-KO, 5xFAD-Trem2-KO; Agilent array: WT and APP/PS1).


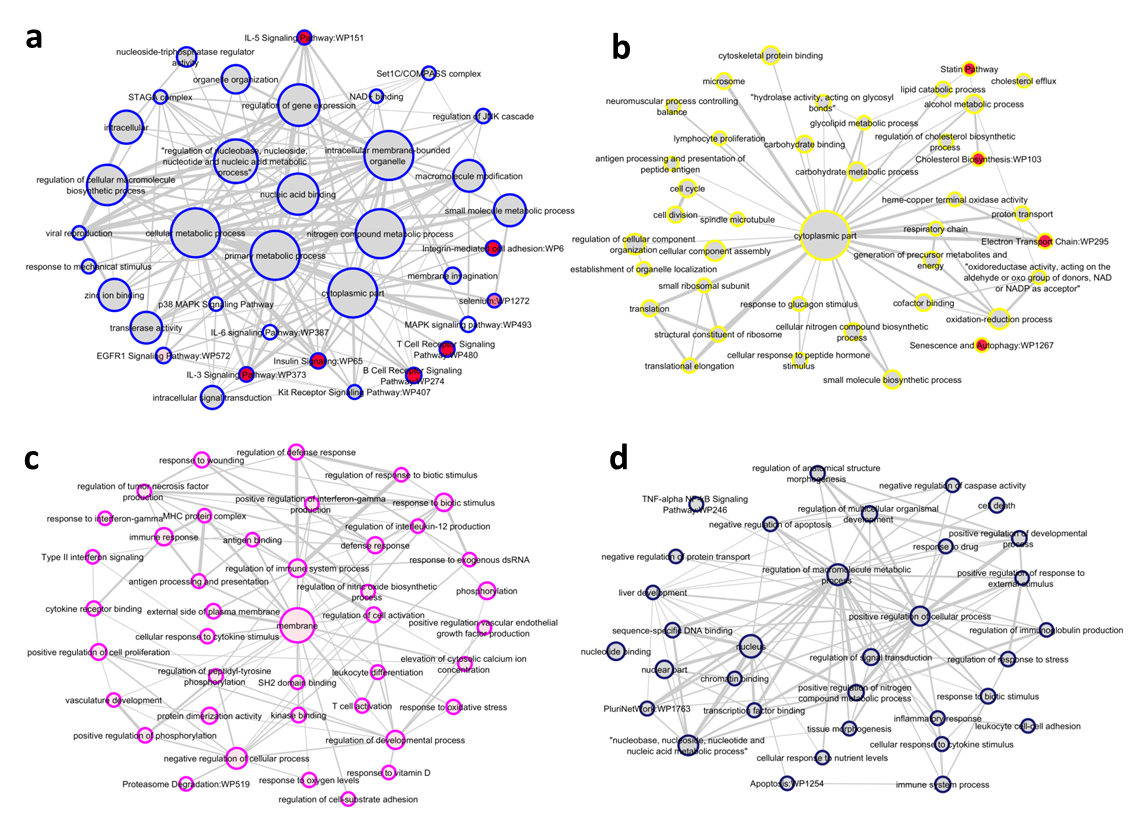


**Figure S2, related to Figure 1. Gene ontology analysis reveals distinct cellular localization and functional profiles of microglial networks in AD.** Results from GO analyses using DAVID were imported into Cytoscape for illustration. Size of each circle represents number of genes within each GO term. Border color indicates module membership and fill color of each circle represents strength of significance of the GO term (red indicates adjusted p<0.01 and grey indicates adjusted p<0.05).


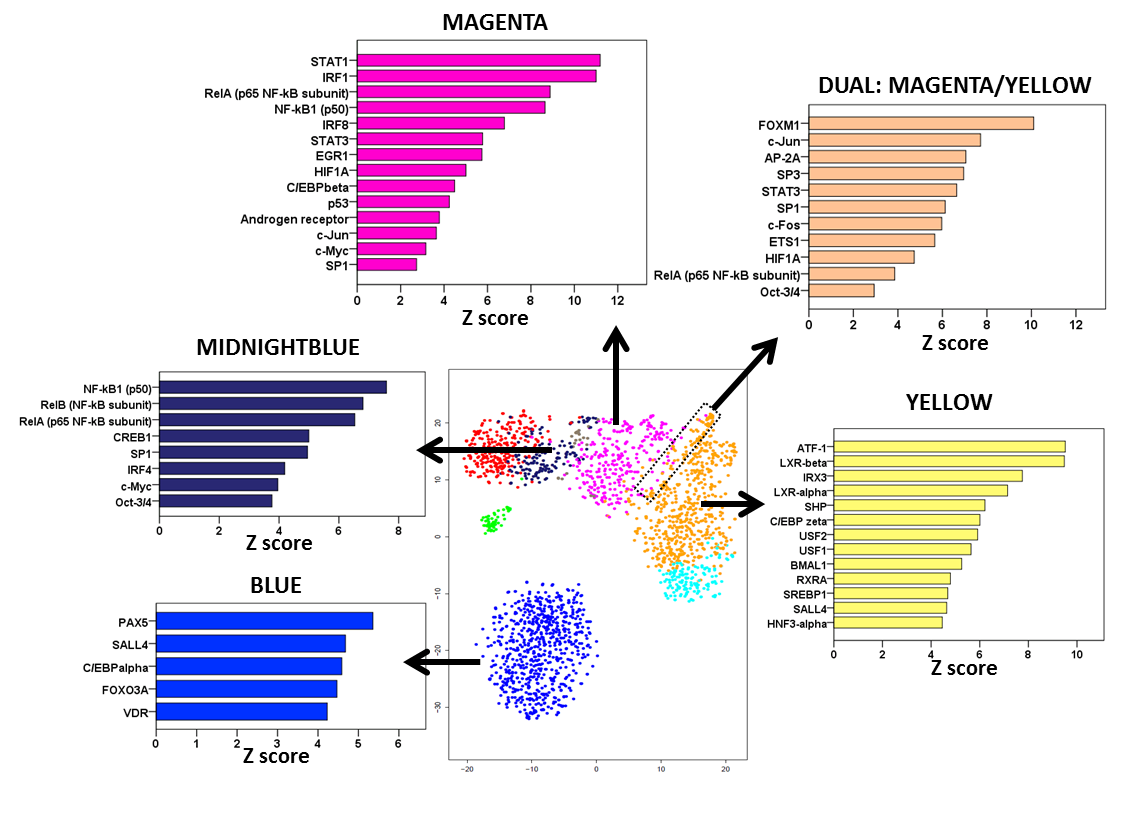


**Figure S3, Related to Figure 1. Identification of transcriptional regulators of AD-associated microglial modules.** Genes specific to Blue, Midnightblue, Magenta and Yellow modules and genes with high membership to both Magenta and Yellow modules (dual members, N=35) were used for identification of upstream transcriptional regulators using Metacore. Z-score represents level of enrichment of genes (Z=1,96 indicates p<0.05) downstream of a transcriptional regulator.

**
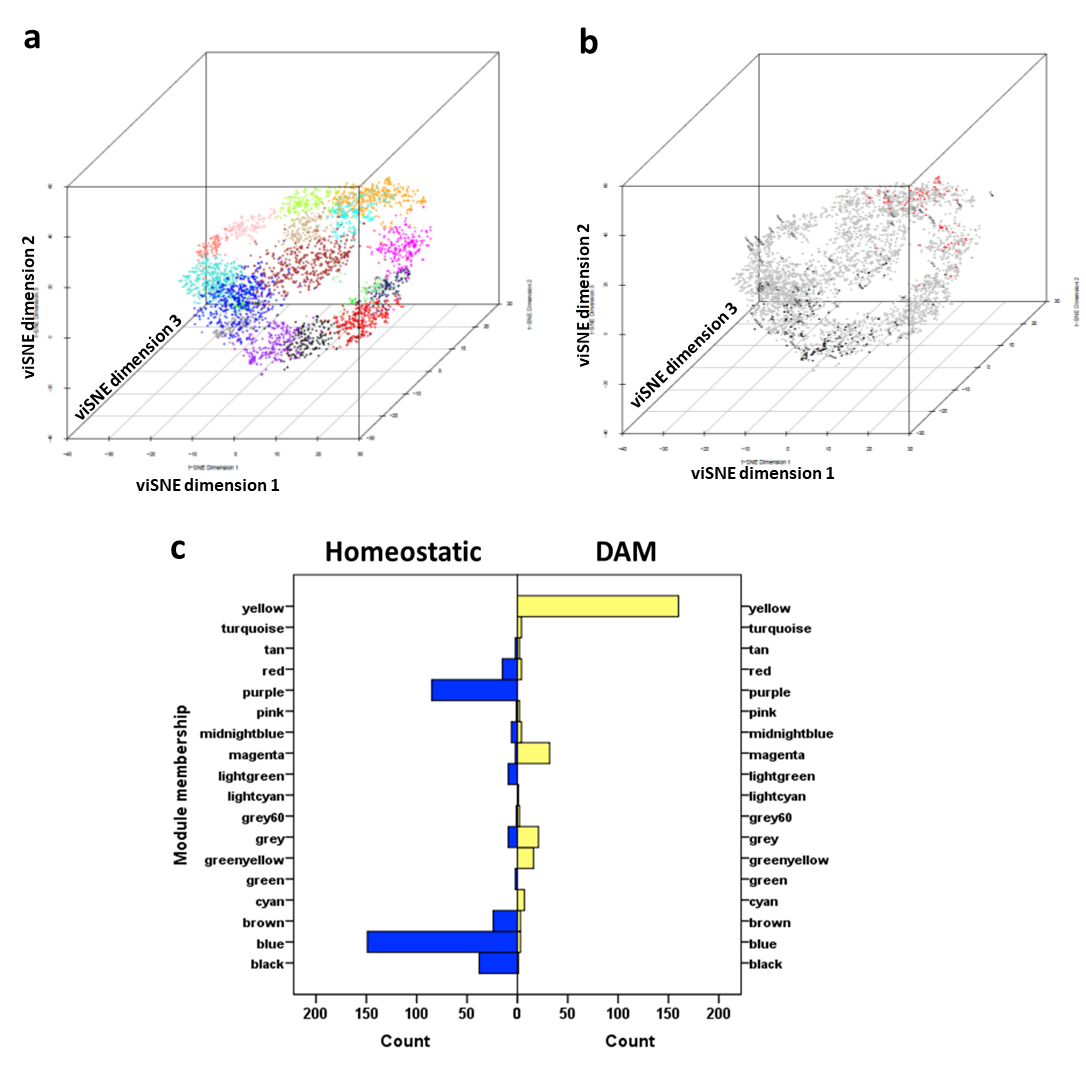
**

**Figure S4, Related to Figure 2. A transcriptomic landscape of microglial activation states in AD.** (a) ViSNE 3D representation of 20 WGCNA-identified gene modules (each module is color coded). For this analysis, WGCNA module members with at least moderate module membership (Kme>=0.65) were included. Three clear potential pathways of microglial activation from homeostatic (Blue) to AD-associated states (Magenta and Yellow) are visible in this representation: a pro-inflammatory pathway (lower band), an anti-inflammatory pathway (middle band) and a third potential pathway (upper band). (b) ViSNE 3D representation of DAM signature genes (red) and homeostatic signature genes (black) identified by single cell RNAseq by Keren Shaul et. al. (Cell, 2017) mapped onto the WGCNA-identified microglial co-expression modules. This shows that DAM genes mapped primarily to Magenta and Yellow modules while homeostatic genes mapped to Blue and Purple modules. (c) DAM signature genes are highly enriched in the Yellow and Magenta modules while Homeostatic signature genes are highly enriched in Blue and Purple microglial modules. The distribution of genes identified by single cell RNAseq as being specific to homeostatic microglia or to DAM (Keren-Shaul, Cell 2017) across microglial transcriptomic modules identified by WGCNA is shown.


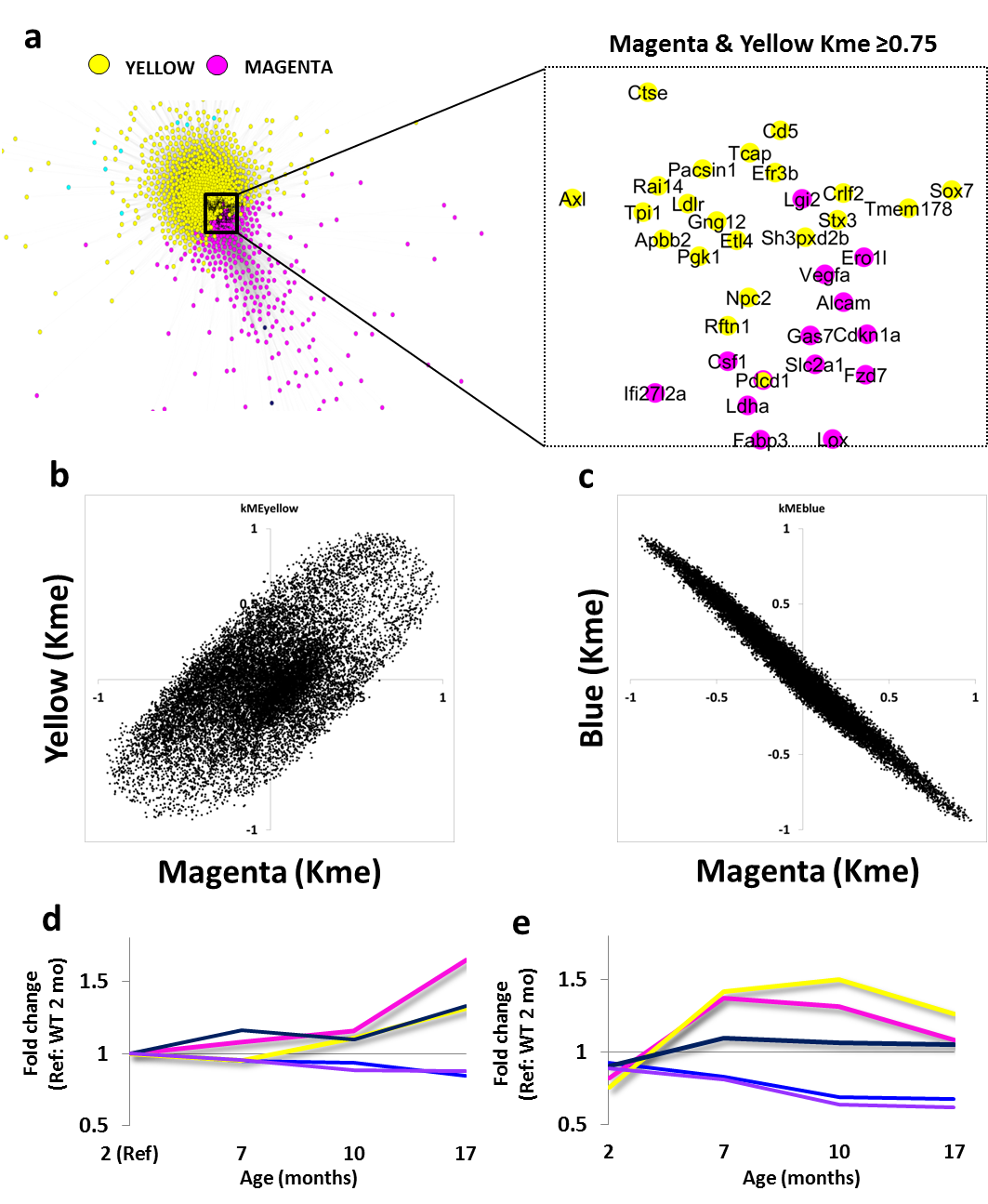


**Figure S5, Related to Figure 2.** **Magenta and Yellow modules likely emerge as distinct subtypes from a common microglial precursor state.** (a) Identification of genes with high level of membership (Kme≥0.75) to both anti-inflammatory Yellow and pro-inflammatory Magenta mouse microglial modules. (b, c) Correlations between each gene’s module membership to Yellow and Magenta (b) or to Blue and Magenta (c) modules are shown. Moderate positive correlation between Magenta and Yellow membership and very strong negative correlation between Blue and Magenta modules are highlighted. In WGCNA, each gene is assigned strength of membership (Kme) to every module (Kme range -1 to +1) and the highest Kme indicates the parent module the gene belongs to. (d, e) Expression of Blue (n=108 genes), Magenta (n=21 genes), Yellow (n=39 genes), Midnightblue (n=12 genes) and Purple (n=52 genes) modules at various ages in WT and APP/PS1 mouse microglia. Data were obtained from a published Nanostring transcriptomics analysis of purified microglia from WT and APP/PS1 mouse brains (aged 2-17 months) (Krasermann et al 2017). Gene-level expression data were first normalized to expression in 2 mo old WT mice. Genes were then assigned module membership after cross-referencing with our mouse microglial WGCNA and genes with Kme ≥0.5 were identified. Each module’s expression was calculated as the median of all normalized expression of all module members and the trajectory of change of each module was mapped. While the expression of the Blue module decreases with aging in both WT and APP/PS1 mice, the Magenta and Yellow modules show early upregulation only in APP/PS1 mice while a gradual upregulation with aging is also seen (also see Table S7).


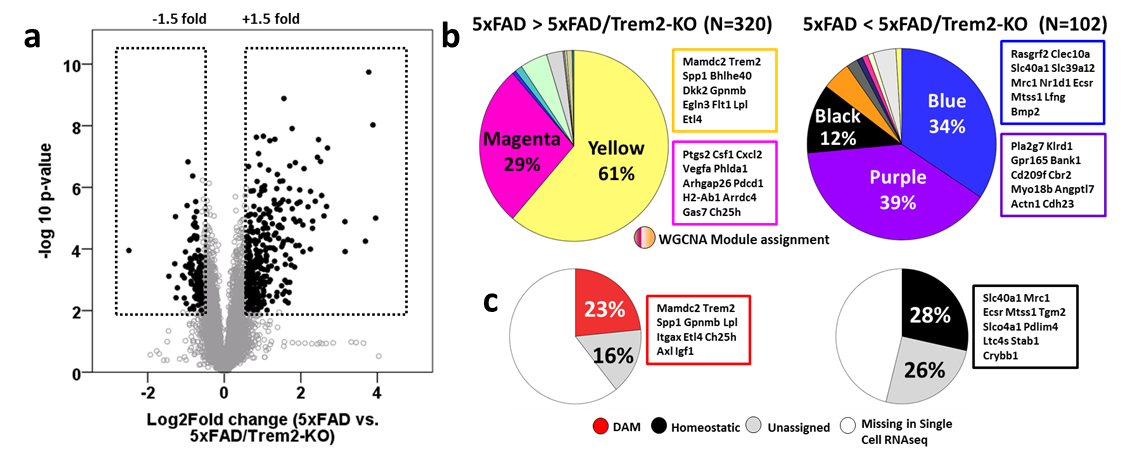


**Figure S6, Related to Figure 2. Pro- and anti-inflammatory DAM networks emerge downstream of the Trem2-mediated immune checkpoint in AD.** (a) Volcano plot of differentially expressed genes in 5xFAD and 5xFAD/Trem2-KO mice. Genes differentially expressed by at least +/- 1.5-fold and p<0.01 are highlighted and represent Trem2-regulated genes. (b) Among Trem2-regulated genes, the Yellow and Magenta modules were highly enriched for genes positively regulated by Trem2 while Blue and Purple modules were highly enriched for genes negatively regulated by Trem2. (c) DAM-specific genes were highly enriched for genes positively regulated by Trem2 while Homeostatic microglial genes were highly enriched for genes negatively regulated by Trem2. Top 10 genes for each group are highlighted.


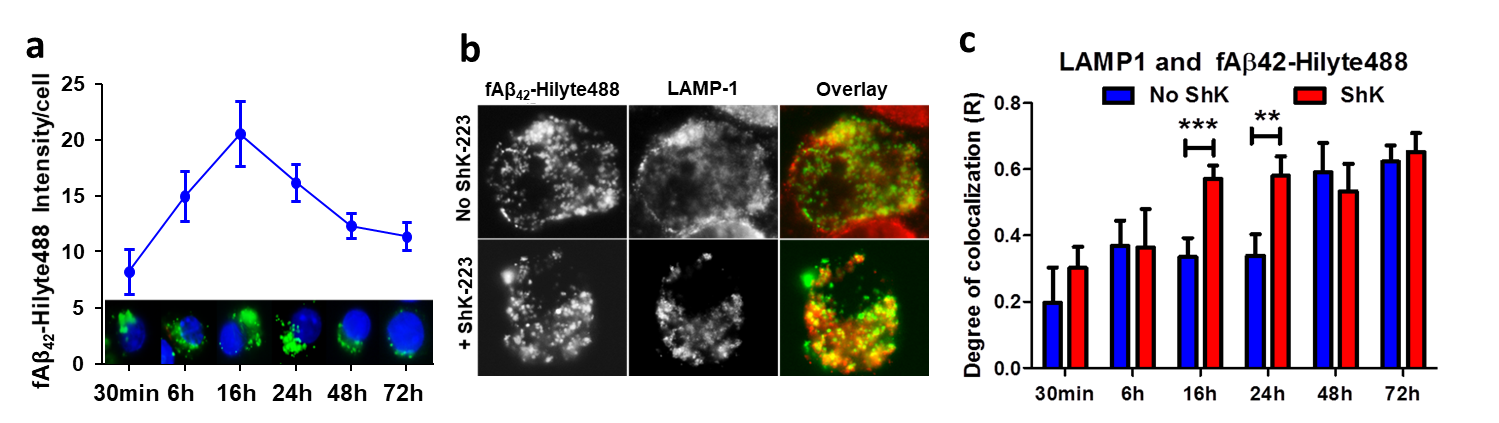


**Figure S7. ShK-223 promotes compartmentalization of Aβ in mature phagolysosomes**

**(a)** Time course of Aβ42 degradation in BV2 microglia. BV2 microglia were loaded with fluorescent Aβ42 fibrils for 30 min and then washed. At 30 min following loading, aggregates were seen being internalized, followed by peak fluorescence and compartmentalization into phagosomes by 16-24 hours after which loss of fluorescence was observed by 72 hours.

**(b, c)** Effect of ShK-223 treatment on compartmentalization of Aβ42 into mature Lamp1-positive phagolysosomes in BV2 microglia. Quantitation of co-localization (mean R coefficient) between LAMP1 (Red) and Aβ42 (Green) was performed at each time point (N=6 independent experiments per condition with 10-12 cells counted per time point).
